# Systemic overexpression of SQSTM1/p62 accelerates disease onset in a SOD1^H46R^-expressing ALS mouse model

**DOI:** 10.1186/s13041-018-0373-8

**Published:** 2018-05-29

**Authors:** Shun Mitsui, Asako Otomo, Masahisa Nozaki, Suzuka Ono, Kai Sato, Ryohei Shirakawa, Hiroaki Adachi, Masashi Aoki, Gen Sobue, Hui-Fang Shang, Shinji Hadano

**Affiliations:** 1 Department of Molecular Life Sciences, Tokai University School of Medicine, Isehara, Kanagawa 259-1193 Japan; 2 The Institute of Medical Sciences, Tokai University, Isehara, Kanagawa 259-1193 Japan; 3 Micro/Nano Technology Center, Tokai University, Hiratsuka, Kanagawa 259-1292 Japan; 4 Department of Anesthesiology, Tokai University School of Medicine, Isehara, Kanagawa 259-1193 Japan; 5 Department of Neurology, University of Occupational and Environmental Health School of Medicine, Kitakyushu, Fukuoka, 807-0804 Japan; 6 Department of Neurology, Tohoku University Graduate School of Medicine, Sendai, Miyagi 980-8575 Japan; 7 Department of Neurology, Nagoya University Graduate School of Medicine, Nagoya, Aichi 466-8550 Japan; 8 Department of Neurology, West China Hospital, Sichuan University, Chengdu, 610041 Sichuan China; 9 Research Center for Brain and Nervous Diseases, Tokai University Graduate School of Medicine, Kanagawa, Isehara, 259-1193 Japan

**Keywords:** Amyotrophic lateral sclerosis, SOD1, SQSTM1/p62, Ubiquitin-positive aggregates

## Abstract

Amyotrophic lateral sclerosis (ALS) is a progressive neurodegenerative disease characterized by a selective loss of upper and lower motor neurons. Recent studies have shown that mutations in *SQSTM1* are linked to ALS. *SQSTM1* encodes SQSTM1/p62 that regulates not only autophagy via the association with MAP1LC3/LC3 and ubiquitinated proteins but also the KEAP1-NFE2L2/Nrf2 anti-oxidative stress pathway by interacting with KEAP1. Previously, we have demonstrated that loss of SQSTM1 exacerbates disease phenotypes in a SOD1^H46R^-expressing ALS mouse model. To clarify the effects of SQSTM1 overexpression in this model, we generated *SQSTM1* and *SOD1*^*H46R*^ double-transgenic (*SQSTM1*;*SOD1*^*H46R*^) mice. *SQSTM1*;*SOD1*^*H46R*^ mice exhibited earlier disease onset and shorter lifespan than did *SOD1*^*H46R*^ mice. Conversely, disease progression after the onset rather slightly but significantly slowed in *SQSTM1*;*SOD1*^*H46R*^ mice. However, there were observable differences neither in the number of Nissl positive neurons nor in the distribution of ubiquitin-positive and/or SQSTM1-positive aggregates between *SOD1*^*H46R*^ and *SQSTM1*;*SOD1*^*H46R*^ mice. It was noted that these protein aggregates were mainly observed in neuropil, and partly localized to astrocytes and/or microglia, but not to MAP2-positive neuronal cell bodies and dendrites at the end-stage of disease. Nonetheless, the biochemically-detectable insoluble SQSTM1 and poly-ubiquitinated proteins were significantly and progressively increased in the spinal cord of *SQSTM1*;*SOD1*^*H46R*^ mice compared to *SOD1*^*H46R*^ mice. These results suggest that overexpression of SQSTM1 in *SOD1*^*H46R*^ mice accelerates disease onset by compromising the protein degradation pathways.

**Electronic supplementary material:**

The online version of this article (10.1186/s13041-018-0373-8) contains supplementary material, which is available to authorized users.

## Introduction

Amyotrophic lateral sclerosis (ALS) is a progressive neurodegenerative disease characterized by a selective loss of upper and lower motor neurons. ALS patients exhibit enhanced tendon reflex caused by loss of upper motor neurons and muscle atrophy, impaired motor control caused by loss of lower motor neurons without sensory disturbance, and ultimately die within 3 to 5 years due to respiratory failure [1]. An approximately 10% of ALS cases is familial and the remaining 90% are sporadic [2]. Although more than 50 different genes that are linked to ALS have been identified, molecular mechanisms by which motor neurons are selectively and progressively degenerated are still unknown [3].

Recently, mutations in the *SQSTM1* gene have been identified in patients with ALS and ALS/frontotemporal dementia (FTD) [4–8]. *SQSTM1* mutations have originally been identified in Paget disease of bone [9]. The *SQSTM1* gene product, sequestosome1 (SQSTM1/p62), is a multi-functional adapter protein. SQSTM1 regulates not only autophagy via the association with microtubule-associated protein 1 light chain 3 (MAP1LC3/LC3) and poly-ubiquitinated proteins [10, 11] but also the Kelch-like ECH-associated protein 1 (KEAP1)-nuclear factor related erythroid 2-related factor 2 (NFE2L2/Nrf2) anti-oxidative stress pathway by interacting with KEAP1 [12]. Indeed, ALS-linked SQSTM1 missense mutant (Leu341Val) [7] showed a decreased affinity to LC3, resulting in impaired autophagy [13]. It has also been shown that SQSTM1 is multiply phosphorylated. Especially, the phosphorylation of SQSTM1 at serine (Ser) 403 (corresponding to mouse Ser405) increases the binding affinity to poly-ubiquitin molecules and enhances the autophagic degradation of poly-ubiquitinated proteins [14, 15]. In addition, it has been reported that the phosphorylation at Ser349 (corresponding to mouse Ser351) enhances the interaction between SQSTM1 and KEAP1, thereby activating the KEAP1-NFE2L2/Nrf2 pathway [16]. With respect to the relationship between SQSTM1 and neurodegenerative diseases, it has been demonstrated that SQSTM1-positive aggregates are accumulated in the spinal cord and brain of ALS and ALS/FTD patients [17–19].

On the other hand, the *SOD1* gene has been identified as a causative gene for an autosomal dominant form of familial ALS in 1993. Thus far, more than 180 different *SOD1* mutations have been identified (http://alsod.iop.kcl.ac.uk/als/). Several transgenic (tg) ALS mouse models expressing different SOD1 mutants have been generated [20]. Among them, *SOD1*^*H46R*^ mutation has originally been identified in Japanese kindred, showing a slow progression of symptoms [21]. *SOD1*^*H46R*^-tg mouse recapitulates many neurological and pathological features observed not in only familial but also in sporadic ALS patients, which include progressive motor neuron degeneration, impaired motor control, and accumulation of SQSTM1 and ubiquitin-positive aggregates in the spinal cord [19, 20, 22, 23].

It has been shown that, in other motor neuron disease (MND) mouse model, i.e., spinal and bulbar muscular atrophy (SBMA) mice, loss of SQSTM1 causes earlier disease onset, whereas overexpression of it ameliorates disease symptoms [24]. We have previously demonstrated that loss of SQSTM1 exacerbates disease phenotypes, including a shorter lifespan, accelerated body weight decline and motor dysfunction, in a mutant *SOD1*^*H46R*^-tg ALS mouse model [25]. These findings suggest a neuroprotective role of SQSTM1 in vivo. However, the effect of SQSTM1 overexpression on the disease onset and progression in mutant SOD1-expressing mice remains unknown.

In this study, to clarify the effect of systemic overexpression of SQSTM1 on the disease onset and progression as well as the accumulation of aggregated proteins in *SOD1*^*H46R*^ mice, we generated SQSTM1-overexpressing *SOD1*^*H46R*^ double-tg (*SQSTM1*;*SOD1*^*H46R*^) mice, and analyzed lifespan, body weight, the number of motor neurons, and the distribution of ubiquitin-positive aggregates in the spinal cord. In addition, we conducted a series of western blot analysis to quantify the amount of expressed protein levels including phosphorylated forms of SQSTM1. We here revealed that overexpression of SQSTM1 accelerated disease onset by compromising the protein degradation pathways in a *SOD1*^*H46R*^-expressing ALS mouse model.

## Methods

### Animals

*SOD1*^*H46R*^-tg mice express human SOD1 carrying the H46R mutation under control of the human *SOD1* promotor [22]. CAG-SQSTM1-HA (*SQSTM1*)-tg mice express human SQSTM1 with hemagglutinin (HA) tag at the C-terminus under control of the CAG [(C) the cytomegalovirus (CMV) early enhancer element, (A) the promoter, the first exon and the first intron of chicken β-actin gene, and (G) the splice acceptor of the rabbit β-globin gene] promoter [24]. Both *SOD1*^*H46R*^-tg and *SQSTM1*-tg mice were backcrossed to 57BL/6 N (B6) mice more than 10 generations, and were maintained as B6 congenic lines. We generated SQSTM1-overexpressing *SOD1*^*H46R*^ mice (*SQSTM1*;*SOD1*^*H46R*^) by crossing male *SOD1*^*H46R*^ mice with female *SQSTM1* mice. The offsprings were genotyped by PCR using genomic DNA extracted from ear tissues. Primers used were as follows; *SOD1*^*H46R*^: hSOD1_ex2: 5′-TCAGAAACTCTCTCCAACTTTGC-3′ and 5′-CAAGTATGGGTCACCAGCAC-3′, hSOD1_ex4: 5′-GGCATCAGCCCTAATCCATC-3′ and 5′-CCGCGACTAACAATCAAAGTG-3′, *SQSTM1-HA*: 5′-AGCTGCCTTGTACCCACATC-3′ and 5′-AGCGTAATCTGGAACATCGT-3′. Mice were housed at 22 °C with a 12 h light-dark cycle. Food and water were fed ad libitum.

### Antibodies

Primary antibodies used for immunohistochemistry were listed as follows; anti-human Ubiquitin (MBL MK-11-3; mouse monoclonal, 1:300), anti-human Ubiquitin (Dako Z0458; rabbit polyclonal, 1:500), anti-human SQSTM1 (Thermo PA5–20839; rabbit polyclonal, 1:300), anti-human p62-C (PROGEN GP62-C; guinea-pig polyclonal, 1:500), anti-rat MAP2 (SIGMA M9942; mouse monoclonal, 1:500), anti-GFAP (NICHIREI 422251; rabbit polyclonal, 1:2), anti-mouse Iba1 (Abcam ab178896; rabbit monoclonal, 1:500), and anti-HA-tag (Cell Signaling 3724; rabbit monoclonal, 1:500) antibodies. Secondary antibodies included Alexa 594 conjugated anti-mouse IgG (Molecular Probes, 1:500), Alexa 594 conjugated anti-Rabbit IgG (Molecular Probes, 1:500), Alexa 594 conjugated anti-guinea-pig IgG (Molecular Probes, 1:500), Alexa 488 conjugated anti-mouse IgG (Molecular Probes, 1:500), and Alexa 488 conjugated anti-Rabbit IgG (Molecular Probes, 1:500) antibodies.

Primary antibodies used for western blotting included anti-human SOD1 (SANTA CRUZ cs-11,407; rabbit polyclonal, 1:15000), anti-human misfolded SOD1 (C4F6; mouse monoclonal, 1:3000) [26], anti-bovine ubiquitin (SANTA CRUZ cs-8017; mouse monoclonal, 1:3000), anti-human SQSTM1 (MBL PM045; rabbit polyclonal, 1:2000), anti-human phosphorylated SQSTM1 (S403) (MBL D343–3; rat monoclonal, 1:3000), anti-mouse phosphorylated SQSTM1 (S351) (MBL PM074; rabbit polyclonal, 1:2000), anti-human LC3 (SIGMA L8918; rabbit polyclonal, 1:5000), anti-human NQO1 (Abcam ab34173; rabbit polyclonal, 1:3000), anti-rabbit GAPDH (MBL M171–3; mouse monoclonal, 1:5000), anti-Actin (SIGMA A5060; rabbit polyclonal, 1:1000), and anti-HA-tag (SANTA CRUZ sc-805; rabbit polyclonal, 1:3000) antibodies. Secondary antibodies included horseradish peroxidase (HRP)-conjugated anti-mouse IgG (Jackson, 1:5000), HRP-conjugated anti-rabbit IgG (GE Healthcare NA934, 1:5000), and HRP-conjugated anti-rat IgG (SANTA CRUZ sc-2006, 1:5000) antibodies.

### Lifespan analysis

Lifespan (endpoint) of tg mice with each genotype (*SOD1*^*H46R*^ and *SQSTM1*;*SOD1*^*H46R*^) were determined by the observations that mice were unable to crawl by their forelimb and to eat/drink by themselves.

### Growth curve analysis

Body weight of mice with each genotype [wild-type (WT), *SQSTM1*, *SOD1*^*H46R*^, and *SQSTM1*;*SOD1*^*H46R*^] was weekly monitored from 5 weeks to a maximum of 32 weeks of age.

### Disease onset and progression analyses

Onset of the disease of mice with each genotype (*SOD1*^*H46R*^ and *SQSTM1*;*SOD1*^*H46R*^) was defined as the point (date) at which body weight was successively decreased from its peak-value in each animal without any increases thereafter. Post-onset survival was defined as the duration from the onset of disease to the endpoint.

### Preparation of paraffin sections

We obtained tissue samples of the spinal cord from *SOD1*^*H46R*^ and *SQSTM1*;*SOD1*^*H46R*^ mice at 16 and 22 weeks of age, and at end-stage. We also obtained the samples from WT and *SQSTM1* mice at 16, 22, and 28 weeks of age. Samples from WT and *SQSTM1* mice at 28 weeks of age were used to compare to those from tg mice at their end-stage. Mice were anesthetized with 4% isoflurane by inhalation and perfused with physiological saline containing 100 U/ml heparin, followed by 4% paraformaldehyde (PFA)/0.1 M phosphate buffer (PB) (pH 7.2). Spinal cord together with the spinal column was removed and fixed with 4% PFA/PB overnight at 4 °C. Lumbar cord (L4-L5) was removed from the spinal column, cut by an approximately 2 mm length (rostral to caudal direction), and post-fixed with 4% PFA/PB overnight at 4 °C. After washing with PB overnight twice, lumbar cord was immersed in 100% methanol for 1 h followed by soaking to fresh methanol overnight. Lumbar cord was further immersed in methanol:chloroform = 1:1 solution for 30 min, and in 100% chloroform for 2 h followed by the treatment with fresh chloroform overnight. The resulting lumbar cord samples were embedded in paraffin. Paraffin-embedded transverse sections at a thickness of 6 μm were prepared by microtome (LEICA RM2165) for Nissl staining and immunohistochemistry.

### Nissl staining

Lumbar cord paraffin sections from mice with four different genotypes at 16 and 22 weeks of age (WT, *SQSTM1*, *SOD1*^*H46R*^, and *SQSTM1*;*SOD1*^*H46R*^), and at end-stage (*SOD1*^*H46R*^ and *SQSTM1*;*SOD1*^*H46R*^) or 28 weeks of age (WT and *SQSTM1*), were treated with xylene (5 min × 3 times) and ethanol (5 min × 3 times), and hydrated. Slices were permeabilized in 1% Triton X-100/phosphate buffered saline (PBS) for 15 min and stained with 50 μl of NeuroTrace green fluorescent Nissl stains (ThermoFisher N21480) (1:500)/PBS for 25 min. Slices were washed with PBS for 2 h and mounted by VECTASHIELD Mounting Medium with DAPI for nuclear staining. A total of 5 representative Nissl staining images of every tenth serial sections from each sample was captured by fluorescence microscope (KEYENCE BZ-9000).

### Counting the number of large Nissl-positive neurons

Large Nissl-positive neurons, representing motor neurons, were identified by using Dynamic cell count (KEYENCE). In brief, tones of the captured images were binarized, and the area covering 600 pixels (px) × 400 px corresponding to anterior horn of the spinal cord was selected. Automatic cell separation procedures (setting resolution; 14, threshold; 45 with excluding cells whose cross-sectional areas were less than 240 px) were executed. Neurons identified were defined as large Nissl-positive neurons and counted. The numbers of large Nissl-positive neurons from 5 representative images from each sample were summed.

### Immunohistochemistry

Spinal cord sections were deparafinized in xylene (5 min × 3 times) and ethanol (5 min × 3 times), and were hydrated. Sections were heated by microwave oven in 300 ml of 0.1 M citric acid (pH 5.0) for 15 min. After cooling at room temperature (RT), sections were washed with PBS and blocked with 50 μl of 0.1% Triton X-100/5% normal goat serum (NGS)/PBS for 30 min. Sections were incubated with primary antibody in 50 μl of the antibody diluted solution (0.05% Triton X-100, 2% NGS/PBS) overnight at 4 °C. Sections were washed with PBS for 2 h and incubated with secondary antibody in 50 μl of the antibody diluted solution for 2 h. Sections were washed with PBS for 1 h and mounted using VECTASHIELD Mounting Medium with DAPI. Signals were analyzed by fluorescence microscope (KEYENCE BZ-710).

### Preparation of tissue samples for western blot analysis

To analyze the expression and distribution of transgene product; i.e., SQSTM1-HA, we obtained tissue samples from WT and *SQSTM1* mice at 16 weeks of age. Tissues including the liver, skeletal muscle, olfactory bulb, cerebral cortex, hippocampus, cerebellum, and whole spinal cord were removed, immediately frozen on dry ice, and stored at − 80 °C until use. To analyze the expression of particular proteins of interest in the spinal cord, we obtained the samples from mice with four different genotypes (WT, *SQSTM1*, *SOD1*^*H46R*^, and *SQSTM1*;*SOD1*^*H46R*^) at 16 and 22 weeks of age, and at end-stage (*SOD1*^*H46R*^ and *SQSTM1*;*SOD1*^*H46R*^) or 28 weeks of age (WT and *SQSTM1*). The whole spinal cord was removed from each mouse, immediately frozen on dry ice, and stored at − 80 °C until use.

### Preparation of protein samples

Spinal cord tissues were weighted and homogenized in 2 weight-volume (mg/μl) of PBS by sonication (Output 2, 10–20 times, BRANSON Sonifier 450). One-hundred microliters of homogenates and 700 μl of Lysis buffer A’ [25 mM Tris-HCl (pH 7.5), 50 mM NaCl, 1% Triton X-100, Complete Protease Inhibitor Cocktail (Roche), Phosphatase inhibiter Cocktail I (SIGMA)] were mixed, followed by centrifugation at 23,000×*g* for 20 min at 4 °C. Supernatant was collected as 1% Triton X-100 soluble fraction. The insoluble pellet was washed by mixing with 600 μl of Lysis buffer A’ and centrifuged again, and resulting pellet was suspended in 400 μl of Lysis buffer B′ [25 mM Tris-HCl (pH 7.5), 50 mM NaCl, 5% sodium dodecyl sulfate (SDS)], sonicated (Output 2, 5–10 times), and used as 1% Triton X-100 insoluble fraction. Protein concentration was determined by Micro BCA (Thermo, MicroBCA Protein Assay kit #23235). Each protein fraction was diluted by the corresponding lysis buffer and adjusted its concentration at 0.75 μg/μl. One-hundred microliters of diluted samples were mixed with 50 μl of 3xSDS buffer [187 mM Tris-HCl (pH 6.8), 6% SDS, 30% glycerol, 0.015% bromophenol blue (BPB), 15% 2-melcaptoethanol] and heated for 5 min at 95 °C.

### Western blot analysis

Equal amount of proteins was subjected to SDS-polyacrylamide gel electrophoresis (PAGE) (Wako Supersep™Ace, 5–20% 17well) in SDS-PAGE buffer [25 mM Tris-HCl, 192 mM glycine, 0.1% (*w*/*v*) SDS]. Precision Plus Protein standards dual color standard (BIO-RAD 1610374) was used as molecular weight markers. Proteins were electro-transferred onto polyvinylidene fluoride (PVDF) membrane (Merckmillipore Immobilon-P) in the transfer buffer [25 mM Tris-HCl, 192 mM glycine, 20% (w/v) methanol]. The membranes were blocked with 0.5% skimmed milk/TBST [20 mM Tris-HCl (pH 7.5), 150 mM NaCl, 0.1% Tween20] for 1 h at RT. When using anti-phosphorylation SQSTM1 antibody, membranes were blocked with 50% blocking one (Wako)/TBST for 30 min at RT. Membranes were incubated with the primary antibody in TBST overnight at 4 °C. After washing with TBST (10 min, 6 times), membranes were incubated with HRP-conjugated secondary antibody in TBST for 2 h at RT. After washing with TBST, signals were visualized by Immobilon (Merck Millipore) and detected by Ez-Capture MG (ATTO).

### Quantification of the signal intensities on western blotting

We conducted a quantitative analysis of the signal intensities on western blotting to determine the expression levels of proteins of interest in 1% Triton X-100 soluble and insoluble factions from mice with four different genotypes (WT, *SQSTM1*, *SOD1*^*H46R*^, and *SQSTM1*;*SOD1*^*H46R*^) at 16 and 22 weeks of age, and at end-stage (*SOD1*^*H46R*^ and *SQSTM1*;*SOD1*^*H46R*^) or 28 weeks of age (WT and *SQSTM1*). The signal intensities were quantified by using CS Analyzer Ver3.0 (ATTO) and were normalized by the levels of glyceraldehyde 3-phosphate dehydrogenase (GAPDH) for soluble fractions or β-actin for insoluble fractions.

### Statistical analysis

All statistical analyses were conducted using Prism 5 (Graph pad). Data for lifespan, onset of disease, and post-onset survival between *SOD1*^*H46R*^ and *SQSTM1*;*SOD1*^*H46R*^ mice were compared using Kaplan-Meier survival analysis with Log-rank test. Statistical significances for body weight, the number of spinal motor neurons, and quantitative western blotting data between groups were evaluated by One-way or Two-way ANOVA with Bonferroni post hoc tests. A *p*-value < 0.05 was considered as reaching statistical significance.

## Results

### Expression and tissue distribution of SQSTM1-HA in SQSTM1 transgenic mice

*SQSTM1*-tg mouse carries the expression-cassette that can express the C-terminally HA-tagged human SQSTM1 (SQSTM1-HA) protein under control of the CAG promoter (Additional file 1: Figure S1a) [24]. To determine the distribution and expression levels of SQSTM1-HA in *SQSTM1* mice, we prepared protein samples from the liver, skeletal muscle, olfactory bulb, cerebral cortex, hippocampus, cerebellum, and spinal cord from WT and *SQSTM1* mice, and performed western blotting using anti-SQSTM1 and anti-HA antibodies. Mouse endogenous SQSTM1 was detected in all tissues examined with highest in the cerebral cortex and hippocampus (Additional file 1: Figure S1b, see WT). The CAG promoter-driven human SQSTM1-HA was highly expressed in skeletal muscle, and also detected in the central nervous system including the spinal cord, while it was undetectable in the liver (Additional file 1: Figure S1b).

### Overexpression of SQSTM1 accelerates body weight declines in SOD1^H46R^ mice

**Fig. 1:**
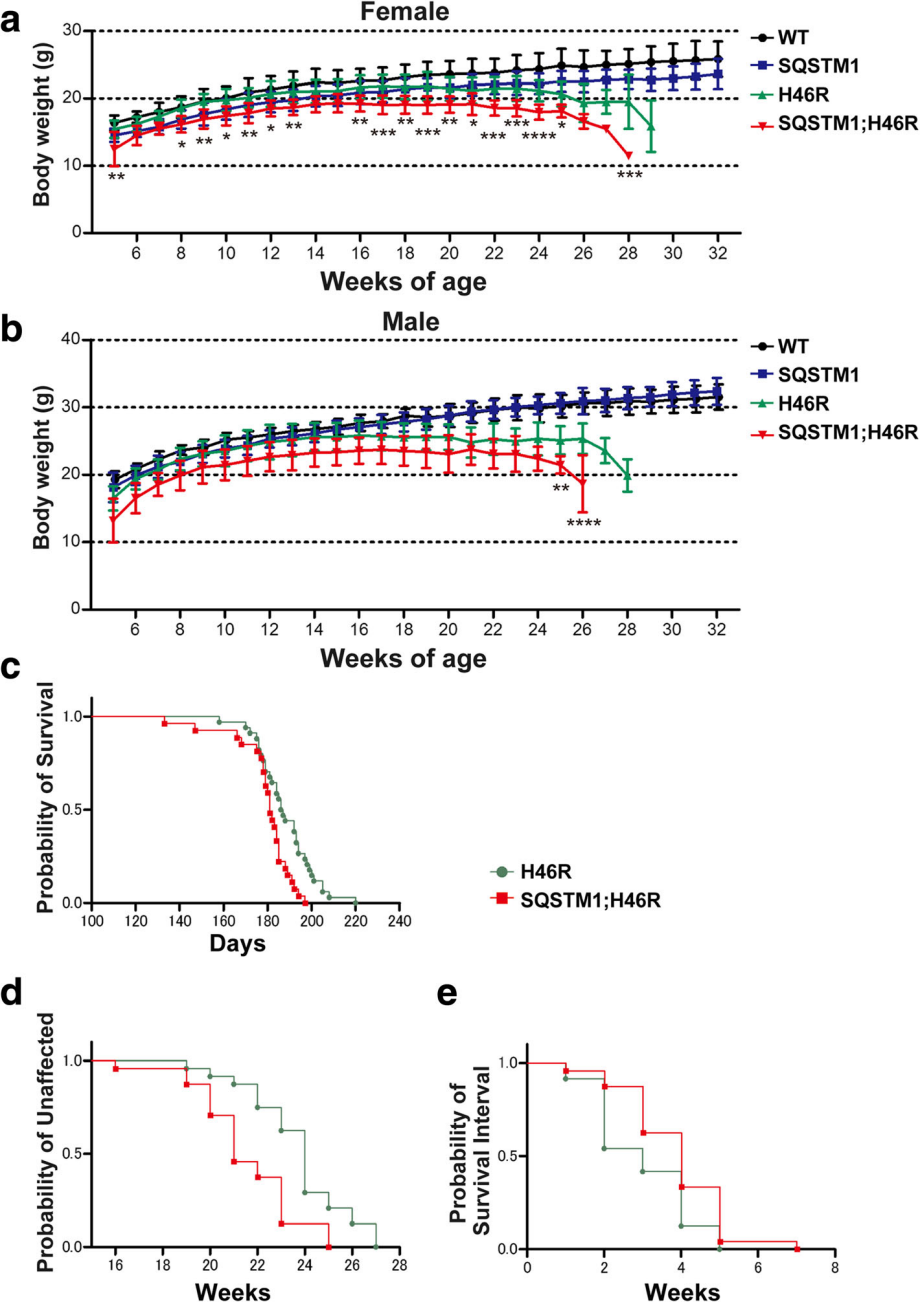
Analysis of lifespan, disease onset, and progression in *SQSTM1;SOD1*^*H46R*^ mice. **a** Growth curve in female mice with four different genotypes [wild-type (WT; *n* = 8–12), *SQSTM1* (SQSTM1; *n* = 9–12), *SOD1*^*H46R*^ (H46R; *n* = 2–12), and *SQSTM1*;*SOD1*^*H46R*^ (SQSTM1;H46R; *n* = 1–12)]. **b** Growth curve in male mice with four different genotypes [WT; *n* = 4–12, SQSTM1; *n* = 11–12, H46R; *n* = 2–12, and SQSTM1;H46R; *n* = 4–12]. **a, b** Body weight of *SQSTM1*;*SOD1*^*H46R*^ mice was significant lower than that of *SOD1*^*H46R*^ mice (*p < 0.05, **p < 0.01, ***p < 0.001, ****p < 0.0001). There are no significantly differences in body weight between WT and SQSTM1 mice. Values were mean ± s.d.. Statistical significance was evaluated by one-way ANOVA with Bonferroni’s post hoc test. **c** Lifespan analysis of *SOD1*^*H46R*^ mice (H46R; *n* = 31) and *SQSTM1*;*SOD1*^*H46R*^ mice (SQSTM1;H46R; *n* = 28). Kaplan-Meier analysis demonstrated that lifespan in *SQSTM1*;*SOD1*^*H46R*^ mice (mean ± s.d.; 179.3 ± 2.6 days) was significantly shorter than that in *SOD1*^*H46R*^ mice (189.3 ± 2.2 days) (Log-rank test; *p* = 0.0013). **d** Onset of the disease in *SOD1*^*H46R*^ (H46R; *n* = 24) and *SQSTM1*;*SOD1*^*H46R*^ (SQSTM1;H46R; *n* = 24) mice. Onset of disease is defined as the turning-point of body weight successive reduction. Onset in SQSTM1;SOD1^H46R^ mice (mean ± s.d.; 21.5 ± 2.1 weeks) was significantly earlier than that in SOD1^H46R^ mice (23.8 ± 2.1 weeks) (Log-rank test; *p* = 0.0007). **e** Survival after the onset of disease (post-onset survival interval) in *SOD1*^*H46R*^ (H46R; *n* = 24) and *SQSTM1*;*SOD1*^*H46R*^ (SQSTM1;H46R; *n* = 24) mice. Survival after the onset is defined as the period between the onset of disease and end-point. Post-onset survival interval in *SQSTM1*;*SOD1*^*H46R*^ mice (mean ± s.d.; 3.9 ± 1.3 weeks) was significantly longer than that in *SOD1*^*H46R*^ mice (3.0 ± 1.1 weeks) (Log-rank test; *p* = 0.0314)

We performed growth curve analysis of WT, *SQSTM1*, *SOD1*^*H46R*^, and *SQSTM1*;*SOD1*^*H46R*^ mice. Both WT and *SQSTM1* mice were viable for at least 32 weeks of age. Throughout the experimental periods, body weights of *SQSTM1* mice tended to be lower than those of WT mice in female (Fig. 1a). Body weights of female *SQSTM1*;*SOD1*^*H46R*^ mice were significantly lower than those of *SOD1*^*H46R*^ mice at 5, 8–13, 16–25, and 28 weeks of age (Fig. 1a). In male, body weights of *SQSTM1;SOD1*^*H46R*^ mice were significantly lower than those of *SOD1*^*H46R*^ mice at 25 and 26 weeks of age (Fig. 1b). These results indicate that overexpression of SQSTM1 in *SOD1*^*H46R*^ mice accelerates weight loss.

### Overexpression of SQSTM1 shortens lifespans in SOD1^H46R^ mice

In the previous study, we have shown that loss of SQSTM1 significantly shortens lifespans in *SOD1*^*H46R*^ mice [25]. Further, it has been reported that loss of SQSTM1 shortens lifespan while overexpression of it prolongs lifespan in SBMA mice [24]. To clarify the effect of overexpression of SQSTM1 on the onset and progression in an ALS mouse model (*SOD1*^*H46R*^ mice), we performed lifespan analysis using *SOD1*^*H46R*^ and *SQSTM1*;*SOD1*^*H46R*^ mice. Since there were no gender differences of lifespans in B6 congenic *SOD1*^*H46R*^ mice [27], we used the gender-combined data. Kaplan-Meier survival analysis revealed that the lifespan of *SQSTM1*;*SOD1*^*H46R*^ mice (179.3 ± 2.6 days) was significantly shorter than that of *SOD1*^*H46R*^ mice (189.3 ± 2.2 days) (Fig. 1c; Log-rank test; *p* = 0.0013).

### Overexpression of SQSTM1 accelerates onset of disease but slightly extends post-onset survival in SOD1^H46R^ mice

Previously, it has been reported that onset of disease can be estimated by the turning-point of the body weight from gain to loss in mutant SOD1-tg mice [27–29]. Growth curve analysis revealed that onset of disease in *SQSTM1*;*SOD1*^*H46R*^ mice (21.5 ± 2.1 weeks) was significantly earlier than those of *SOD1*^*H46R*^ mice (23.8 ± 2.1 weeks) (Fig. 1d and Table 1; Log-rank test; *p* = 0.0007). To investigate the difference in disease progression between *SOD1*^*H46R*^ and *SQSTM1*;*SOD1*^*H46R*^ mice, we analyzed the survival interval after disease onset (post-onset survival). Contrary to the disease onset, survival interval after the onset of disease in *SQSTM1*;*SOD1*^*H46R*^ mice (3.9 ± 1.3 weeks) was slightly but significantly extended when compared to *SOD1*^*H46R*^ mice (3.0 ± 1.1 weeks) (Fig. 1e and Table 1; Log-rank test; *p* = 0.0314).

**Table 1:** Summary of survival and onset point analysis in *SOD1*^*H46R*^ and *SQSTM1;SOD1*^*H46R*^ mice

Genotype	Number	End point (weeks)	s.d.	Onset (weeks)	s.d.	Post-onset survival interval (weeks)	s.d.
SOD1^H46R^	24	26.7	1.5	23.8	2.1	3.0	1.1
SQSTM1;SOD1^H46R^	24	25.4	1.5	21.5	2.1	3.9	1.3

Number represents the number of mice used in survival and onset analyses. Values represent mean ± s.d. (weeks)

### Progressive decreases in the number of motor neurons in the lumbar spinal cord of SOD1^H46R^-expressing mice

**Fig. 2:**
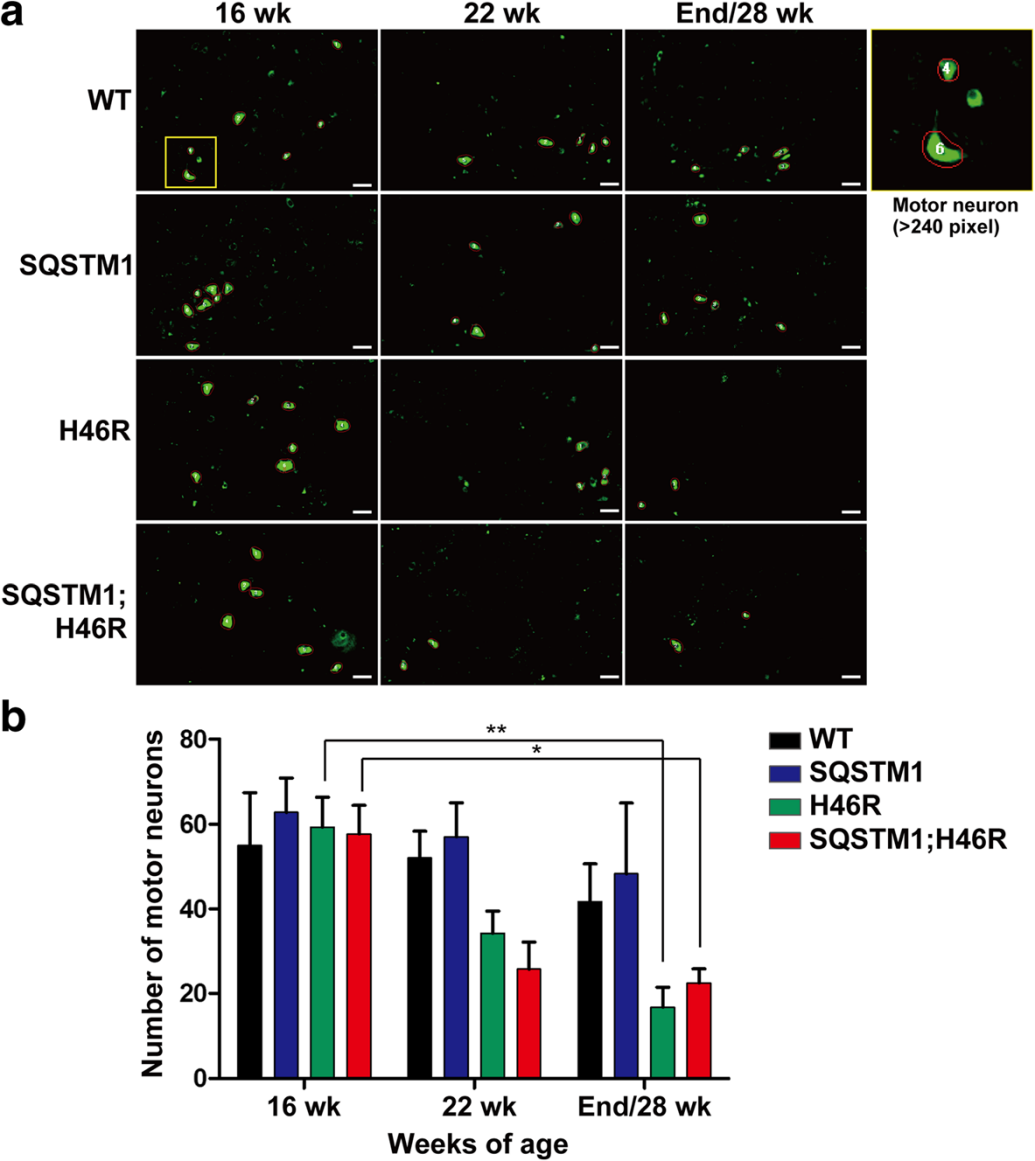
Quantitative analysis of motor neurons in the anterior horn of lumbar spinal cord. **a** Representative images for fluorescence Nissl staining in the anterior horn. Nissl positive neurons were detected in the anterior horn of lumbar spinal cord (L4–5) from the mice with four different genotypes; wild-type (WT), *SQSTM1*, *SOD1*^*H46R*^ (H46R), and *SQSTM1*;*SOD1*^*H46R*^ (SQSTM1;H46R) at 16 and 22 weeks of age (wk), and at end-stage (H46R and SQSTM1;H46R) or at 28 wk. (WT and *SQSTM1*). Cells covering the area of over 240 square pixels (px), corresponding to large motor neurons, are surrounded by red circles, and counted. Scale bars = 50 μm. **b** Number of large Nissl-positive neurons in the anterior horn of lumbar spinal cord. Vertical axis represents the cumulative number of motor neurons identified in 5 representative Nissl staining images of every tenth serial sections from each sample (*n* = 3–4, *SOD1*^*H46R*^ and *SQSTM1*;*SOD1*^*H46R*^ mice at 22 wk. and end-stage; *n* = 4, others; *n* = 3). Values are mean ± s.e.m.. Statistical significance was evaluated by two-way ANOVA with Bonferroni’s post hoc test (*p < 0.05, **p < 0.01)

To investigate whether earlier disease onset observed in *SQSTM1*;*SOD1*^*H46R*^ mice was associated with motor neuron loss in the spinal cord, we performed Nissl staining and counted the number of large neurons in the anterior horn of lumbar spinal cord. In this analysis, mice with four different genotypes; WT, *SQSTM1*, *SOD1*^*H46R*^, and *SQSTM1*;*SOD1*^*H46R*^ mice at 16 and 22 weeks of age, and at end-stage (*SOD1*^*H46R*^ and *SQSTM1*;*SOD1*^*H46R*^) or 28 weeks of age (WT and *SQSTM1*), were used. The numbers of large Nissl-positive neurons were progressively decreased both in *SOD1*^*H46R*^ and *SQSTM1*;*SOD1*^*H46R*^ mice compared to WT and *SQSTM1* mice (Fig. 2 and Additional file 2: Fig. S2a). However, there were no significant differences in the numbers of Nissl positive large neurons between groups both at 22 weeks of age and the end-stage, suggesting that decrease in the number of motor neurons did not explain the reasons why overexpression of SQSTM1 not only accelerates the onset of disease but also slows survival after the onset in *SOD1*^*H46R*^ mice.

### Accumulation of ubiquitin/SQSTM1-positive aggregates and the activation of glial cells in SOD1^H46R^-expressing mice

**Fig. 3:**
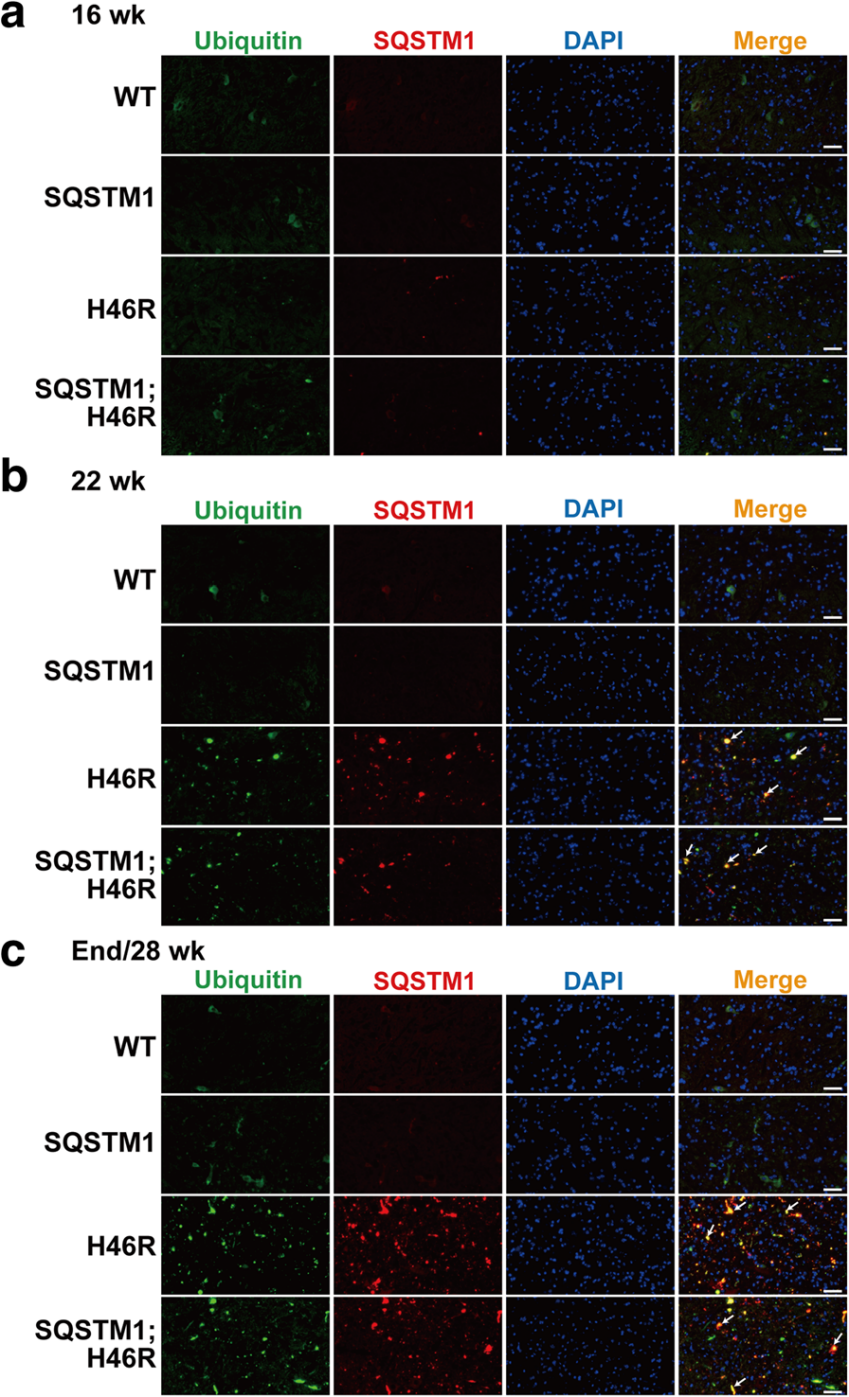
Progressive accumulation of ubiquitin/SQSTM1-positive aggregates in the anterior horn of lumbar spinal cord. **a-c** Representative images of double immunostaining with Ubiquitin (green) and SQSTM1 (red) in the lumbar spinal cord (L4–5) from wild-type (WT), *SQSTM1* (SQSTM1), *SOD1*^*H46R*^ (H46R) and *SQSTM1*;*SOD1*^*H46R*^ (SQSTM1;H46R) mice at 16 weeks of age (wk) (**a**), 22 wk. (**b**), and end-stage (H46R and SQSTM1;H46R) or 28 wk. (WT and SQSTM1) (**c**). The nuclei were counterstained with DAPI (blue). Scale bars = 50 μm. Ubiquitin-positive aggregates and SQSTM1 aggregates were observed in the anterior horn of *SOD1*^*H46R*^ (H46R) and *SQSTM1*;*SOD1*^*H46R*^ (SQSTM1;H46R) mice at 22 wk. and end-stage. Arrows indicate ubiquitin-positive aggregates that are colocalized with SQSTM1

**Fig. 4:**
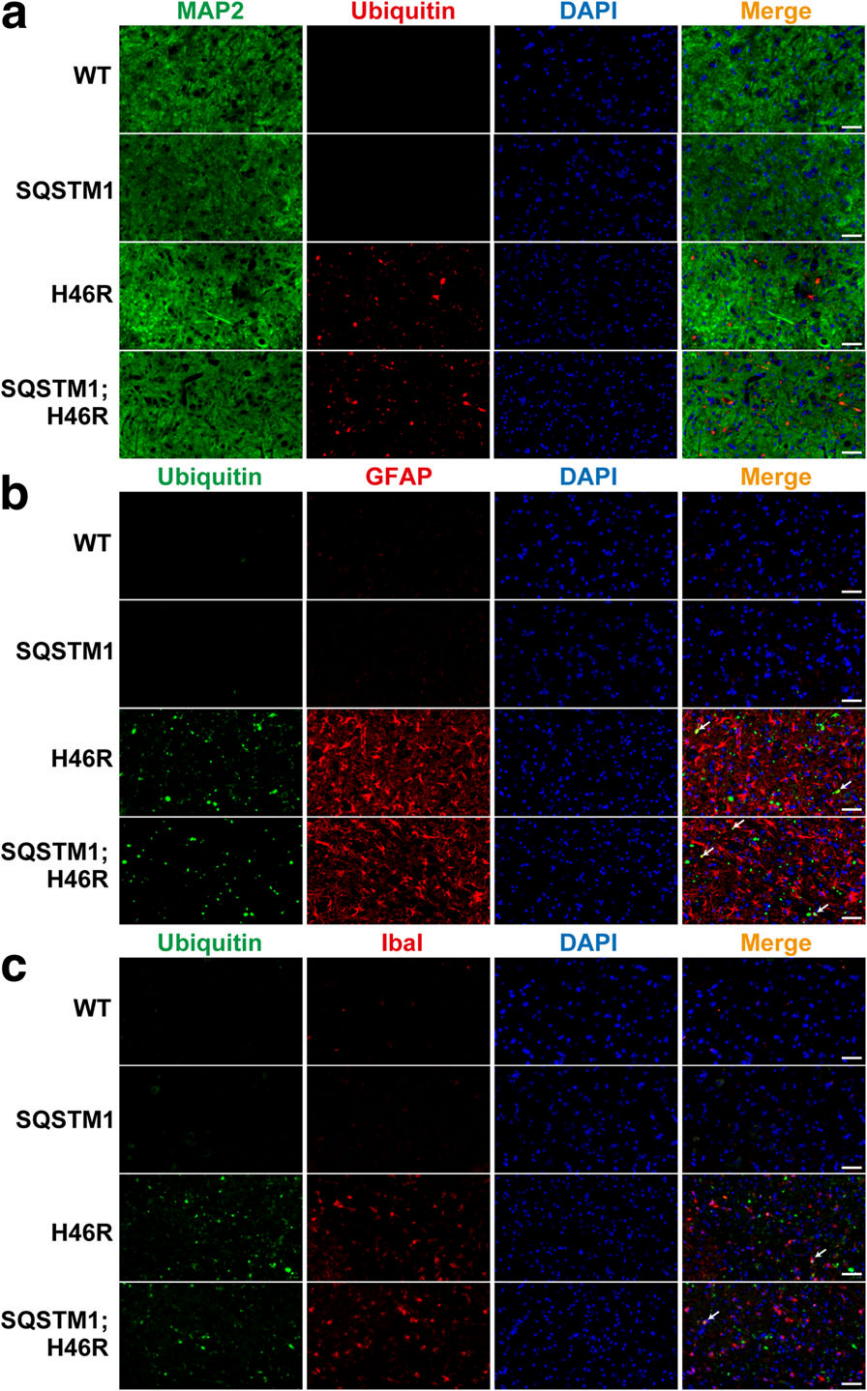
Extensive accumulation of ubiquitin-positive aggregates in the spinal cord from *SOD1*^*H46R*^-expressing mice at end-stage. **a-c** Representative images of double immunostaining with MAP2 (green; neuron marker) and Ubiquitin (red) (**a**), Ubiquitin (green) and GFAP (red; astrocyte marker) (**b**), Ubiquitin (green) and Iba1 (red; microglia marker) (**c**) in the lumbar spinal cord (L4–5) from wild-type (WT) and *SQSTM1* (SQSTM1) at 28 weeks of age, *SOD1*^*H46R*^ (H46R) and *SQSTM1*;*SOD1*^*H46R*^ (SQSTM1;H46R) mice at end-stage. The nuclei were counterstained with DAPI (blue). Scale bars = 50 μm. **b** Arrows indicate colocalization of ubiquitin-positive aggregates with GFAP-positive astrocytes. **c** Arrows indicate colocalization of ubiquitin-positive aggregates with Iba1-positive microglia

**Fig. 5:**
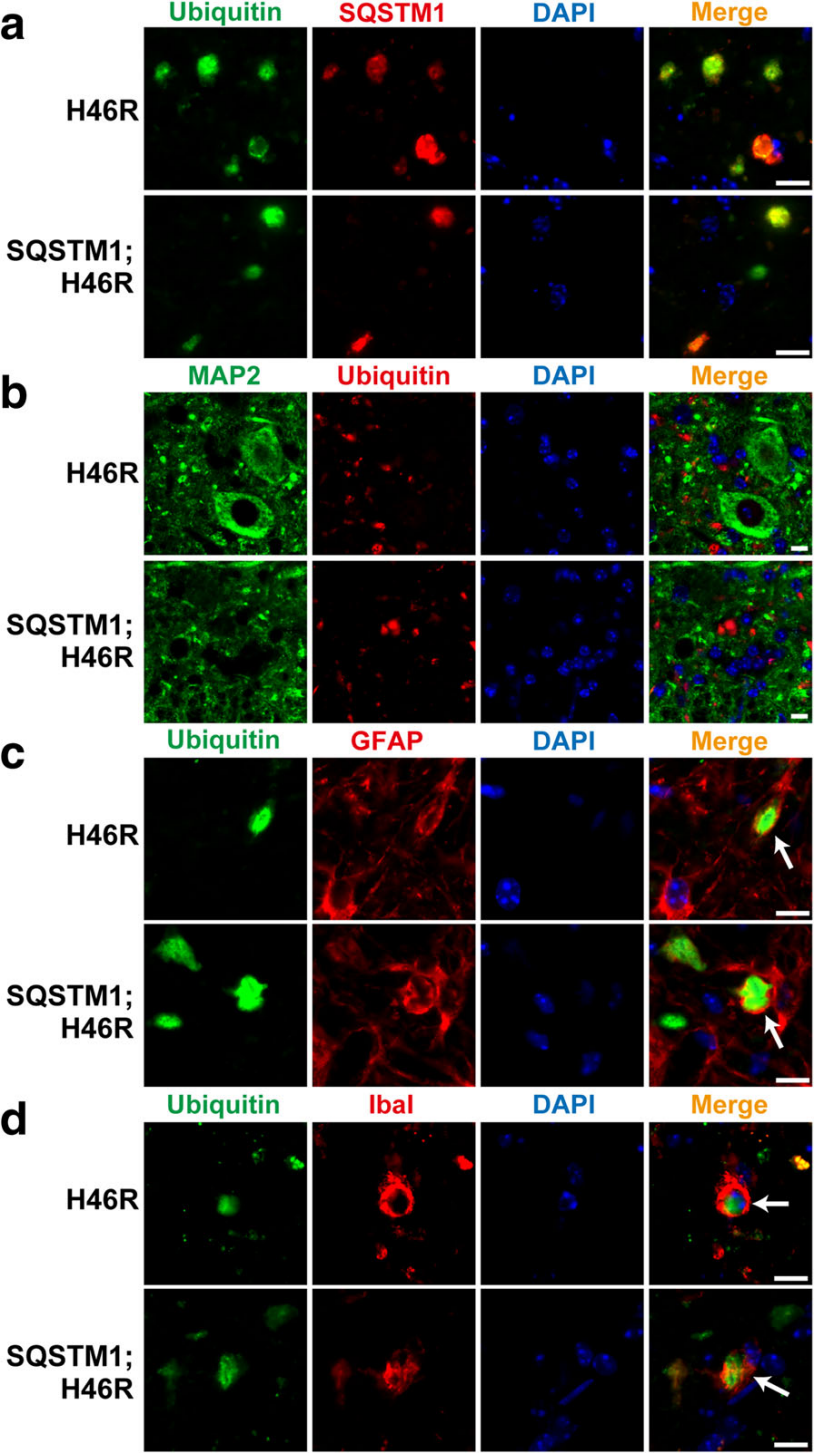
High-magnification images for the distribution of ubiquitin-positive aggregates in the spinal cord from *SOD1*^*H46R*^-expressing mice. **a-d** Representative images of double immunostaining with Ubiquitin (green) and SQSTM1 (red) (**a**), MAP2 (green; neuron marker) and Ubiquitin (red) (**b**), Ubiquitin (green) and GFAP (red; astrocyte marker) (**c**), Ubiquitin (green) and Iba1 (red; microglia marker) (**d**) in the lumbar cord (L4–5) from *SOD1*^*H46R*^ (H46R) and *SQSTM1*;*SOD1*^*H46R*^ (SQSTM1;H46R) mice at end-stage. The nuclei were counterstained with DAPI (blue). Scale bars = 10 μm. **c** Arrows indicate that ubiquitin-positive aggregates are present in GFAP-positive astrocytes. **d** Arrows indicate that ubiquitin-positive aggregates are surrounded by microglia

SQSTM1 acts as an adaptor protein that directly binds to poly-ubiquitinated proteins destined for degradation in autophagy [10]. It has been reported that accumulation of ubiquitin- and SQSTM1-positive aggregates is observed not only in ALS patients but also in a *SOD1*^*H46R*^ ALS mouse model [22], and that proliferation of astrocytes and the activation of microglia also occur in *SOD1*^*H46R*^ mice as disease progresses. To clarify the effect of SQSTM1 overexpression on the localization of ubiquitin-/SQSTM1-positive aggregates and the activation of glial cells, we conducted immunohistochemical analysis of ubiquitin and SQSTM1 in the lumbar cord (L4–5) from WT, *SQSTM1*, *SOD1*^*H46R*^, and *SQSTM1*;*SOD1*^*H46R*^ mice at 16 and 22 weeks of age, and at end-stage (*SOD1*^*H46R*^ and *SQSTM1*;*SOD1*^*H46R*^) or 28 weeks of age (WT and *SQSTM1*) (Fig. 3 and Additional file 2: Figure S2b). We also conducted immunohistochemistry for microtubule-associated protein 2 (MAP2), glial fibrillary acidic protein (GFAP), and ionized calcium binding adaptor molecule 1 (Iba1) using mice with four different genotypes at end-stage (*SOD1*^*H46R*^ and *SQSTM1*;*SOD1*^*H46R*^) or 28 weeks of age (WT and *SQSTM1*). MAP2, GFAP, and Iba1 were used as cellular markers for neuronal soma and dendrite, astrocytes, and microglia, respectively (Figs. 4 and 5, and Additional file 2: Figure S2c).

Double immunostaining of ubiquitin and SQSTM1 revealed that a progressive accumulation of ubiquitin- and SQSTM1-positive aggregates was observed in the spinal cord of *SOD1*^*H46R*^-expressing mice (*SOD1*^*H46R*^ and *SQSTM1*;*SOD1*^*H46R*^) as disease progressed (Fig. 3 and Additional file 2: Figure S2b). It was also noted that a majority of the ubiquitin-positive signals was colocalized with SQSTM1 (Figs. 3 and 5a). Further, SQSTM1-HA aggregates were immunohistochemically detected in *SQSTM1;SOD1*^*H46R*^ mice at 22 weeks and end-stage by using the anti-HA-tag antibody (Additional file 3: Figure S3), indicating that overexpressing SQSTM1 was also progressively accumulated in the spinal cord as was endogenous SQSTM1. However, there were no apparent differences in the amount and/or number of ubiquitin-/SQSTM1-positive aggregates between *SOD1*^*H46R*^ and *SQSTM1*;*SOD1*^*H46R*^ mice.

Double immunostaining of ubiquitin and MAP2 revealed that ubiquitin-positive aggregates were not present in MAP2-positive neuronal cell bodies or dendrites in the lumbar cord of *SOD1*^*H46R*^-expressing mice (Figs. 4a and 5b and Additional file 2: Figure S2c). On the other hand, double immunostaining of ubiquitin and GFAP demonstrated that the number of GFAP-positive astrocytes was significantly increased in *SOD1*^*H46R*^-expressing mice (Fig. 4b and Additional file 2: Figure S2c), and that small numbers of ubiquitin-positive aggregates were localized to astrocytes (Figs. 4b and 5c; arrows). Activation of Iba1-positive microglia was also observed in *SOD1*^*H46R*^-expressing mice (Fig. 4c and Additional file 2: Figure S2c). Ubiquitin-positive aggregates that were surrounded by microglia were occasionally observed (Fig. 5d; arrows). However, there were no differences in the levels of glial cell activation between *SOD1*^*H46R*^ and *SQSTM1*;*SOD1*^*H46R*^ mice.

Together, these results indicate that ubiquitin-/SQSTM1-positive aggregates were mainly localized to neuropil and occasionally to astrocytes and/or microglia but not to the cell body of neurons at the end-stage of *SOD1*^*H46R*^-expressing mice. However, there were no discernible differences in the distribution and the degree of colocalization of ubiquitin-positive aggregates to glial cells between *SQSTM1*;*SOD1*^*H46R*^ and *SOD1*^*H46R*^ mice.

### Overexpression of SQSTM1 increases insoluble poly-ubiquitinated proteins in the spinal cord of SOD1^H46R^ mice

To biochemically investigate the accumulation of SOD1, SQSTM1, and ubiquitin, we extracted Triton X-100 soluble and insoluble proteins from the spinal cord of mice with four different genotypes; WT, *SQSTM1*, *SOD1*^*H46R*^, and *SQSTM1*;*SOD1*^*H46R*^ mice at 16 and 22 weeks of age, and at end-stage (*SOD1*^*H46R*^ and *SQSTM1*;*SOD1*^*H46R*^) or at 28 weeks of age (WT and *SQSTM1*) and performed western blot analysis, followed by the quantification of signal intensities.

**Fig. 6:**
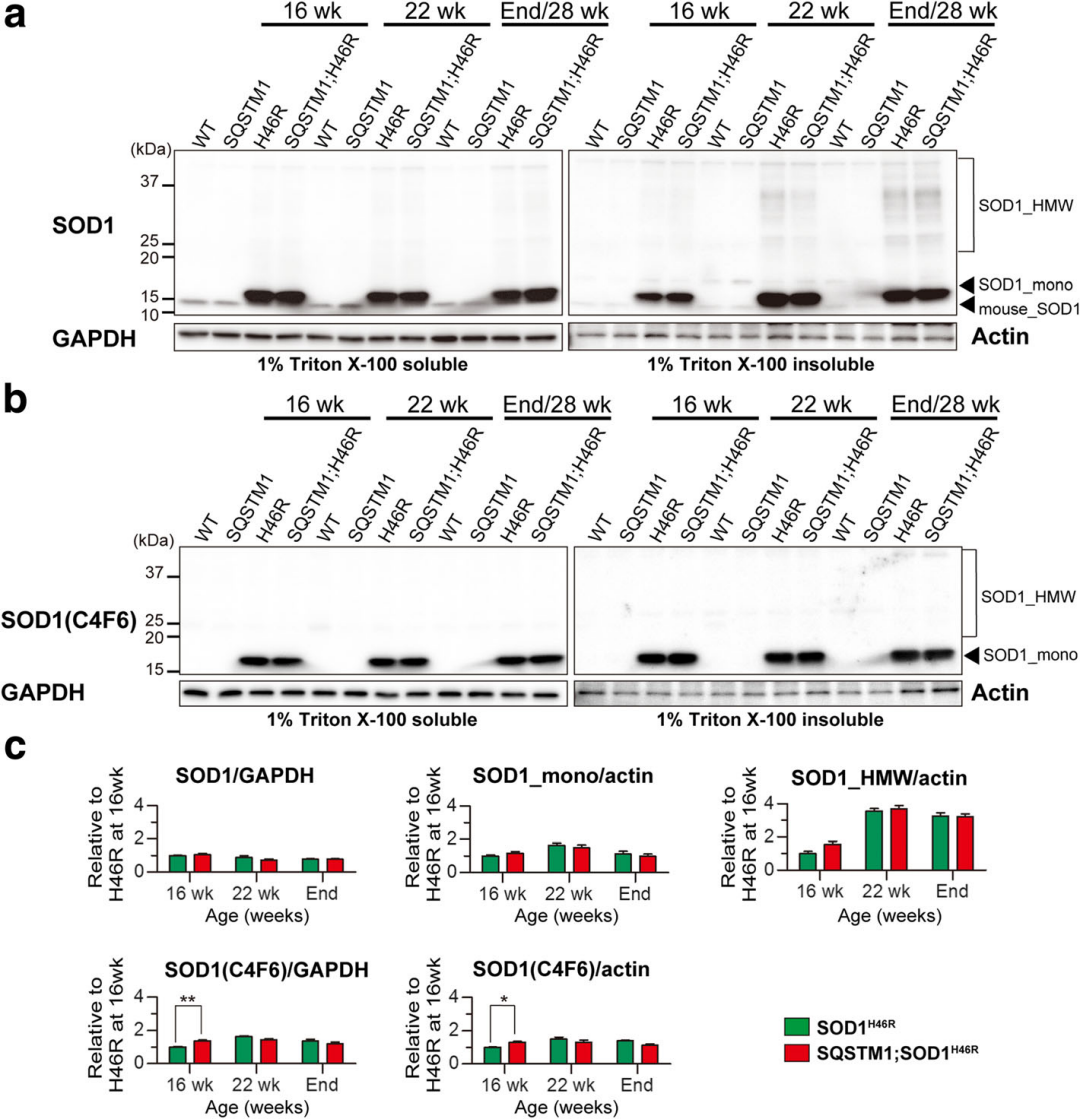
Overexpression of SQSTM1 increases misfolded SOD1 in the spinal cord of *SOD1*^*H46R*^-expressing mice. **a** Western blot analysis of SOD1. **b** Western blot analysis of misfolded SOD1. **a, b** The spinal cord samples were obtained from wild-type (WT), *SQSTM1* (SQSTM1), *SOD1*^*H46R*^ (H46R), and *SQSTM1*;*SOD1*^*H46R*^ (SQSTM1;H46R) mice at 16 weeks of age (wk), 22 wk., and end-stage (H46R and SQSTM1;H46R) or 28 wk. (WT and SQSTM1). Two fractions; 1% Triton X-100 soluble and 1% Triton X-100 insoluble/5%SDS soluble fractions were analyzed. SOD1_mono and SOD1_HMW represent monomeric and high molecular-weight forms of SOD1, respectively. SOD1(C4F6) represents misfolded SOD1. GAPDH and β-actin (Actin) were used for a loading control in Triton X-100-soluble and -insoluble fractions, respectively. **c** Quantification of 1% Triton X-100 soluble SOD1, insoluble SOD1_mono, insoluble SOD1_HMW, soluble misfolded SOD1, and insoluble misfolded SOD1. Values are mean ± s.e.m. (*n* = 4) in an arbitrary unit relative to H46R mice at 16 weeks of age. Signal intensities were normalized by the levels of GAPDH (soluble fractions) and Actin (insoluble fractions). Statistical significance was evaluated by two-way ANOVA with Bonferroni’s post hoc test (comparisons between different genotypes in the same age; *p < 0.05, **p < 0.01)

Although a progressive accumulation of insoluble high-molecular weight SOD1 (SOD1_HMW) was observed both in *SOD1*^*H46R*^ and *SQSTM1;SOD1*^*H46R*^ mice as disease progressed (Fig. 6a and Additional file 4: Figure S4c), quantitative analyses showed no significant differences of the amount of insoluble SOD1_HMW and monomer SOD1 (SOD1_mono) between *SOD1*^*H46R*^ and *SQSTM1*;*SOD1*^*H46R*^ mice (Fig. 6c). Further, we detected misfolded SOD1 molecules using anti-human misfolded SOD1 antibody (C4F6) [26]. Misfolded monomeric SOD1 was observed both in *SOD1*^*H46R*^ and *SQSTM1;SOD1*^*H46R*^ mice, but not in WT and *SQSTM1* mice (Fig. 6b). Quantitative analyses showed that both soluble and insoluble misfolded SOD1 in *SQSTM1;SOD1*^*H46R*^ mice at 16 weeks of age were significantly higher than those in *SOD1*^*H46R*^ mice, while there were no differences between two groups at the later stages of disease (22 weeks of age and end-stage) (Fig. 6c and Additional file 5: Figure S5).

**Fig. 7:**
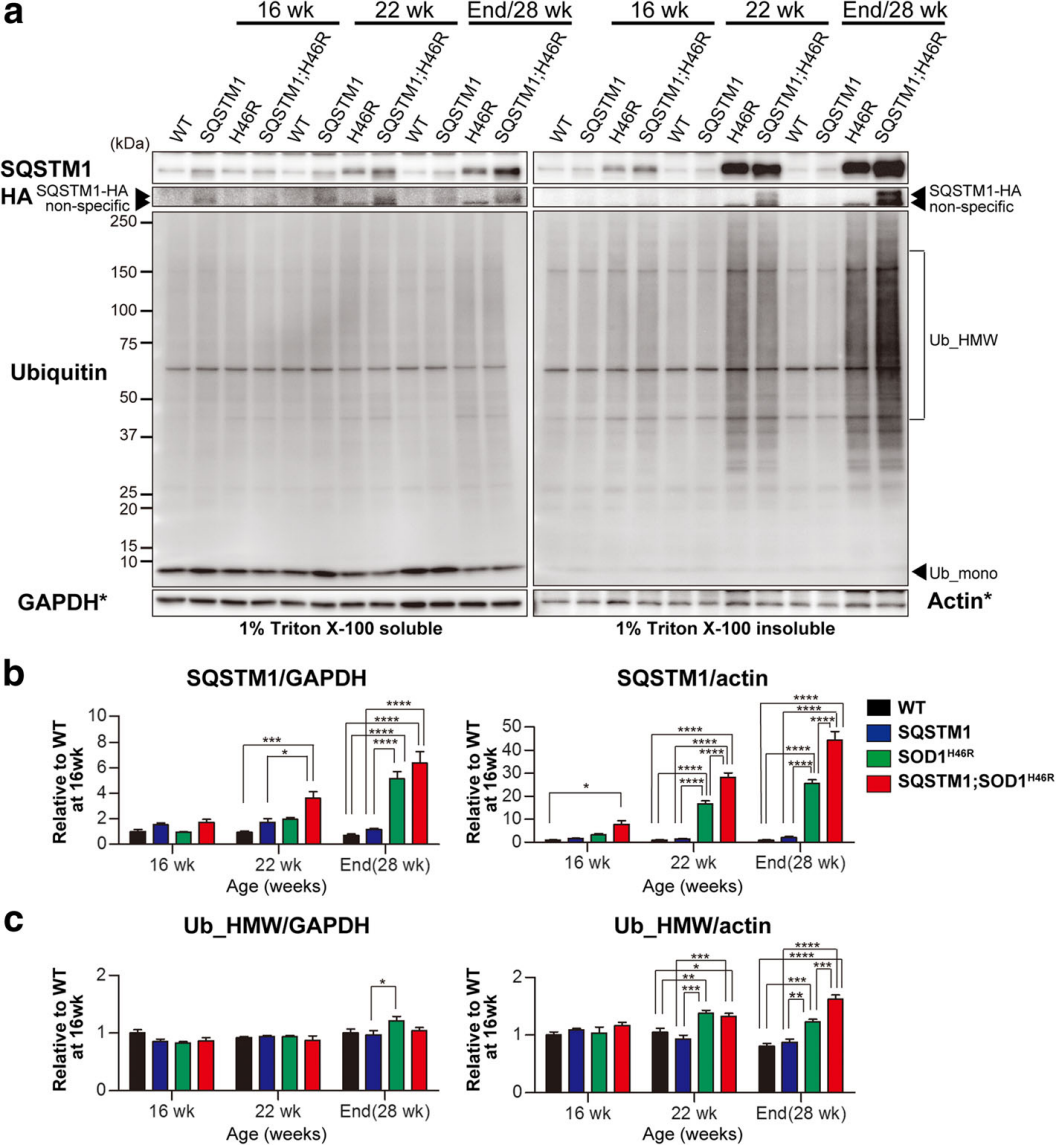
Overexpression of SQSTM1 increases the insoluble poly-ubqiuitinated proteins in the spinal cord of *SOD1*^*H46R*^-expressing mice. **a** Western blot analysis of SQSTM1, SQSTM1-HA (HA) and Ubiquitin (Ub) in the spinal cord from wild-type (WT), *SQSTM1* (SQSTM1), *SOD1*^*H46R*^ (H46R), and *SQSTM1*;*SOD1*^*H46R*^ (SQSTM1;H46R) mice at 16 weeks of age (wk), 22 wk., and end-stage (H46R and SQSTM1;H46R) or 28 wk. (WT and SQSTM1). Two fractions; 1% Triton X-100 soluble and 1% Triton X-100 insoluble/5%SDS soluble fractions were analyzed. SOD1_mono and SOD1_HMW represent monomeric and high molecular-weight forms of SOD1, respectively. Ub_mono and Ub_HMW represent monomeric ubiquitin and poly-ubiquitinated proteins, respectively. GAPDH and β-actin (Actin) were used for a loading control in Triton X-100-soluble and -insoluble fractions, respectively. Since the blots used in these western analyses were same as those in Fig. 6a, same images for GAPDH and Actin (asterisks) in Fig. 6a were used again as references. **b** Quantification of soluble and insoluble SQSTM1. **c** Quantification of soluble and insoluble poly-ubiquitinated proteins (Ub_HMW). **b, c** Values are mean ± s.e.m. (*n* = 4) in an arbitrary unit relative to WT mice at 16 weeks of age. Signal intensities were normalized by the levels of GAPDH (soluble fractions) and Actin (insoluble fractions). Statistical significance was evaluated by two-way ANOVA with Bonferroni’s post hoc test (comparisons between different genotypes in the same age; *p < 0.05, **p < 0.01, ***p < 0.001, ****p < 0.0001)

Both soluble and insoluble SQSTM1 were significantly and progressively increased in *SOD1*^*H46R*^ and *SQSTM1*;*SOD1*^*H46R*^ mice as disease progressed (Fig. 7a, b and Additional file 4: Figure S4d and S4e). Compared to *SOD1*^*H46R*^ mice, the accumulated levels of insoluble SQSTM1 in *SQSTM1*;*SOD1*^*H46R*^ mice were much higher at 16 and 22 weeks of age, and at end-stage (Fig. 7b). To distinguish between mouse endogenous and transgene-derived SQSTM1, we performed western blot analysis using anti-HA antibody, and revealed that transgene-encoded SQSTM1-HA was also progressively accumulated as insoluble forms in *SQSTM1*;*SOD1*^*H46R*^ mice (Fig. 7a).

Accumulation of insoluble poly-ubiquitinated proteins that were represented by smear signals was observed in *SOD1*^*H46R*^-expressing mice (Fig. 7a and Additional file 4: Figure S4 g). Although poly-ubiquitinated proteins detected in the spinal cord of *SQSTM1*;*SOD1*^*H46R*^ mice were comparable to those in *SOD1*^*H46R*^ mice at 22 weeks of age, those in *SQSTM1;SOD1*^*H46R*^ mice at end-stage were significantly higher than that in *SOD1*^*H46R*^ mice (Fig. 7a and c).

These results suggest that overexpression of SQSTM1 accelerates the accumulation not only of misfolded SOD1 at the earlier stage, but also of insoluble poly-ubiquitinated proteins at the later stage of disease in the spinal cord of *SOD1*^*H46R*^-expressing mice.

### Overexpression of SQSTM1 increases insoluble phosphorylated SQSTM1 in the spinal cord of SOD1^H46R^ mice

Previous studies have shown that phosphorylation of SQSTM1 at Ser403 regulates degradation of poly-ubiquitinated proteins by autophagy [14], and that protein aggregates containing Ser403-phosphorylated SQSTM1 are enriched in the brain of ALS patients [30]. Further, phosphorylation of SQSTM1 at Ser351 increases the binding of KEAP1 to SQSTM1, thereby activating the NFE2L2/Nrf2 mediated anti-oxidative stress pathway [16]. These phosphorylation sites are conserved between human and mouse and can be detected by specific anti-phospho-SQSTM1 antibodies. To elucidate the effect of SQSTM1 overexpression on autophagy and anti-oxidative stress responses in *SOD1*^*H46R*^ mice, we performed western blot analysis using antibodies to specifically detect Ser403(human)/Ser405(mouse)-phosphorylated SQSTM1, Ser349(human)/Ser351(mouse)-phosphorylated SQSTM1, MAP1LC3/LC3, and NAD(P)H dehydrogenase [quinone] 1 (NQO1) in the spinal cord of WT, *SQSTM1*, *SOD1*^*H46R*^, and *SQSTM1*;*SOD1*^*H46R*^ mice at 16 and 22 weeks of age, and at end-stage (*SOD1*^*H46R*^ and *SQSTM1*;*SOD1*^*H46R*^) or at 28 weeks of age (WT and *SQSTM1*).

**Fig. 8:**
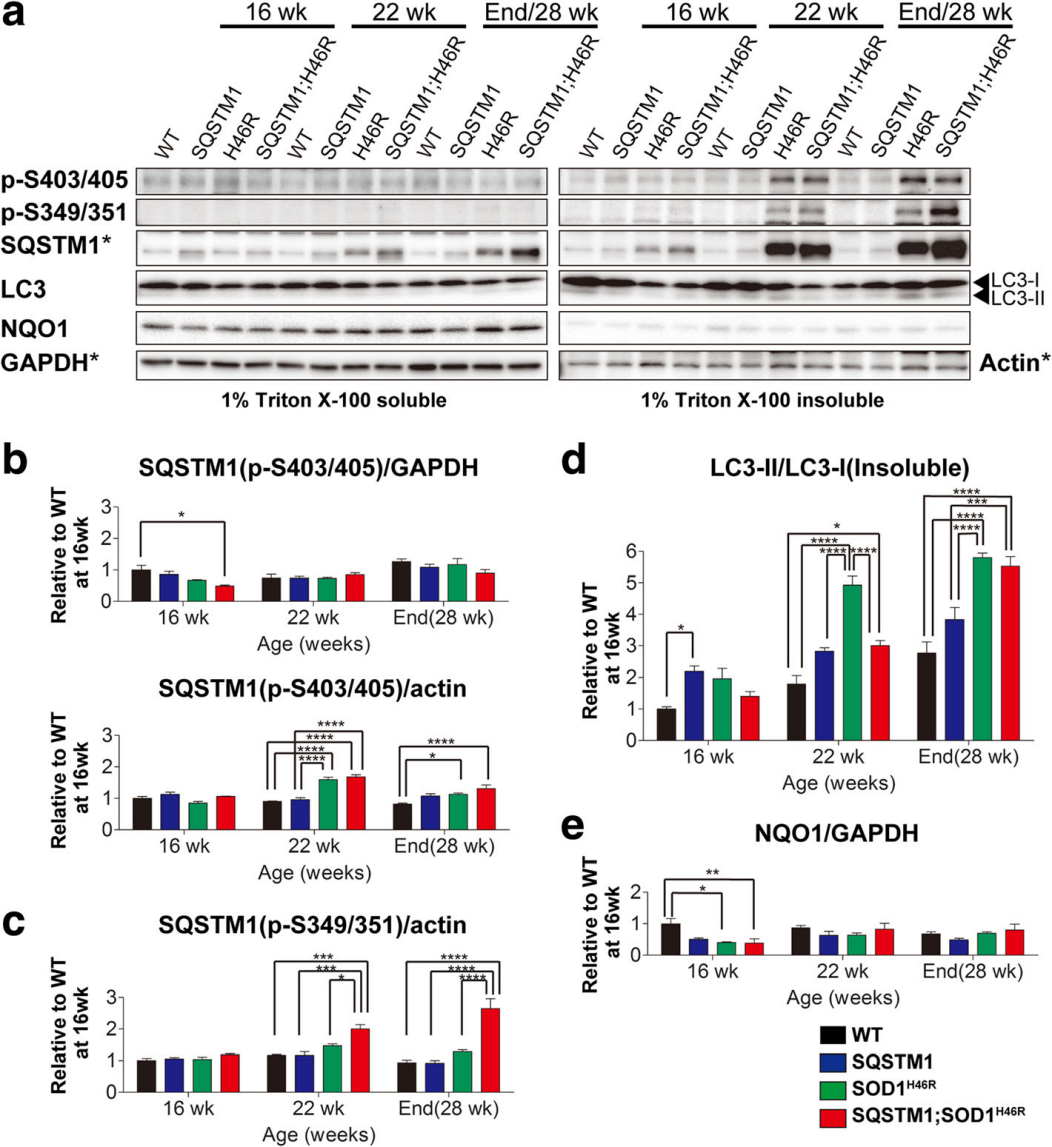
Accumulation of phosphorylated SQSTM1 in the spinal cord of *SOD1*^*H46R*^-expressing mice. **a** Western blot analysis of Ser403(human)/Ser405(mouse)-phosphorylated SQSTM1 (p-S403/405), Ser349(human)/Ser351(mouse)-phosphorylated SQSTM1 (p-S349/351), LC3, and NQO1 in the spinal cord from mice with four different genotypes; wild-type (WT), *SQSTM1* (SQSTM1), *SOD1*^*H46R*^ (H46R), and *SQSTM1*;*SOD1*^*H46R*^ (SQSTM1;H46R) mice at 16 weeks of age (wk), 22 wk., and end-stage (H46R and SQSTM1;H46R) or 28 wk. (WT and SQSTM1). Two fractions; 1% Triton X-100 soluble and 1% Triton X-100 insoluble/5%SDS soluble fractions were analyzed. GAPDH and β-actin (Actin) were used for a loading control in Triton X-100-soluble and -insoluble fractions, respectively. To properly compare the intensities of signals between phosphorylated SQSTM1 and total SQSTM1, the results from same blots for SQSTM1 in Fig. 7a, GAPDH, and Actin (asterisks) in Fig. 6a are used again as references. **b** Quantification of 1% Triton X-100 soluble and insoluble S403/S405-phosphorylated SQSTM1. **c** Quantification of insoluble S349/S351-phosphorylated SQSTM1. **d** Quantification of the LC3-II to LC3-I ratio (LC3-II/LC3-I) in insoluble fraction. **e** Quantification of soluble NQO1. **b-e** Values are mean ± s.e.m. (*n* = 4) in an arbitrary unit relative to WT mice at 16 weeks of age. Signal intensities were normalized by the levels of GAPDH (soluble fractions) and Actin (insoluble fractions). Statistical significance was evaluated by two-way ANOVA with Bonferroni’s post hoc test (comparisons between different genotypes in the same age; *p < 0.05, **p < 0.01, ***p < 0.001, ****p < 0.0001)

The levels of insoluble Ser403/Ser405-phosphorylated SQSTM1 were increased both in *SOD1*^*H46R*^ and *SQSTM1*;*SOD1*^*H46R*^ mice at 22 weeks of age and end-stage compared to WT and *SQSTM1* mice. However, there were no differences in the amount of Ser403/Ser405-phosphorylated SQSTM1 between *SOD1*^*H46R*^ and *SQSTM1*;*SOD1*^*H46R*^ mice, despite that the total amount of SQSTM1 was progressively increased at the end-stage (Figs. 7a, b and 8a, b, and Additional file 6: Figure S6b). Remarkably, Ser349/Ser351-phosphorylated SQSTM1 was specifically detected in insoluble but not in soluble fractions. Further, insoluble Ser349/Ser351-phosphorylated SQSTM1 was significantly increased in *SQSTM1*;*SOD1*^*H46R*^, but not in *SOD1*^*H46R*^, mice as disease progressed (Fig. 8a, c and Additional file 6: Fig. S6d).

The levels of LC3-II in insoluble fraction were progressively increased in *SOD1*^*H46R*^-expressing mice (Fig. 8a and Additional file 6: Figure S6f). The quantitative analysis of the LC3-II to LC3-I ratio (LC3-II/LC3-I), a marker of the autophagosome formation, demonstrated that increase in the levels of LC3-II/LC-I was more prominent in *SOD1*^*H46R*^ mice compared to *SQSTM1*;*SOD1*^*H46R*^ mice (Fig. 8a and d). On the other hand, the levels of NQO1, which was one of the NFE2L2/Nrf2 target proteins, were affected neither by overexpression of mutant SOD1, SQSTM1, nor both of them (Fig. 8a and e, and Additional file 6: Figure S6 g).

These results suggest that overexpression of SQSTM1 affects the autophagy activation and/or the impairment of autophagic degradation of LC3-II positive autophagosomes, whereas it has no impact on the NFE2L2/Nrf2 mediated anti-oxidative stress pathway even in conditions in which the levels of Ser349/Ser351-phosphlylated SQSTM1 is upregulated. It is thus reasonable to assume that phosphorylated SQSTM1 molecules present in the insoluble fraction might be biologically inactive and are present as inert insoluble aggregates in the spinal cord of *SOD1*^*H46R*^ mice.

## Discussion

We have previously shown that loss of SQSTM1 exacerbates disease phenotypes in *SOD1*^*H46R*^ mice [25]. Further, in a SBMA mouse model, loss of SQSTM1 also accelerates the onset of disease, while overexpression of it ameliorates disease phenotypes [24]. These findings suggest a protective role of SQSTM1 in motor neuron degeneration in vivo. In this study, we investigated the effect of systemic overexpression of SQSTM1 on the disease onset and progression in *SOD1*^*H46R*^ mice. Contrary to our expectation, we revealed that systemic overexpression of SQSTM1 resulted in earlier onset and shorter lifespan in a *SOD1*^*H46R*^-expressing ALS mouse model.

It has been reported that phosphorylation at Ser349 (corresponding to mouse Ser351) of SQSTM1 enhances the binding of SQSTM1 to KEAP1 which in turn activates the NFE2L2/Nrf2 pathway and NFE2L2/Nrf2 mediated anti-oxidative stress responses in mice [16]. On the other hand, the previous study on NFE2L2/Nrf2 deficient *SOD1*^*H46R*^ mice has revealed that loss of NFE2L2/Nrf2 affects neither the onset of disease nor the progression of disease [25]. In this study, despite that the accumulation of Ser349/Ser351-phosphorylated SQSTM1 was increased in *SQSTM1*;*SOD1*^*H46R*^ mice at 22 weeks of age and end-stage (Fig. 8a and c), there were no differences in the expression levels of NQO1, which was one of the NFE2L2/Nrf2 downstream targets, between *SOD1*^*H46R*^ and *SQSTM1;SOD1*^*H46R*^ mice. Further, immunohistochemical analysis revealed that ubiquitin/SQSTM1-positive aggregates were not localized in the cell body and dendrite of neurons but mainly to neuropil (Figs. 4 and 5), indicative of dysfunctional SQSTM1 molecules in insoluble aggregates. Taken together, we speculate that progressively-increased Ser349/Ser351-phosphorylated SQSTM1 is physiologically inactive, and thus the NFE2L2/Nrf2 anti-oxidative stress pathway plays a minor role both in the disease onset and progression.

We suppose three possible reasons as to why overexpression of SQSTM1 accelerates the onset of disease in *SOD1*^*H46R*^ mice, unlike in the case of a SBMA mouse model [24]. One possibility is that protein homeostasis (proteostasis) in the spinal cord of *SOD1*^*H46R*^ mice is more severely compromised than those in SBMA mice under conditions in which SQSTM1 is overexpressed. A large number of studies has revealed that the accumulation of aggregated proteins is one of the pathological features of neurodegenerative diseases. Misfolded ubiquitinated proteins are preferentially degraded by the ubiquitin-proteasome system (UPS), but undegradable misfolded proteins by the UPS form aggregates, which in turn are degraded by autophagy [31]. SQSTM1 acts as an autophagy adaptor for the degradation of ubiquitinated proteins [11]. Thus, in addition to the UPS, autophagy is the important intracellular degradation pathway to maintain proteostasis [32], and its impairment results in the accumulation of SQSTM1 and ubiquitinated proteins [10]. Recently, it has been demonstrated that the crossbreeding of conditional *Atg7* knockout mice with *SOD1*^*G93A*^ mice remarkably extends the lifespan, despite that the onset of hindlimb tremor is accelerated [33]. Further, the pharmacological induction of mTOR-independent autophagy has failed to slow the disease progression of *SOD1*^*G93A*^ mice [34]. More importantly, motor neuron-specific disruption of autophagy displays no phenotypic changes in ALS mice, while proteasome inhibition leads to ALS-associated pathology and paralysis [35]. We here showed that misfolded SOD1 in *SQSTM1;SOD1*^*H46R*^ mice at 16 weeks of age were significantly higher than those in *SOD1*^*H46R*^ mice (Fig. 6c). We also demonstrated that the extensive accumulation of insoluble SQSTM1 occurred in *SQSTM1*;*SOD1*^*H46R*^ mice even prior to the onset of disease; i.e., at 16 weeks of age (Fig. 7a and b). Although phosphorylation of human SQSTM1 at Ser403 was known to induce autophagic degradation of ubiquitinated proteins [14], the levels of Ser403 (corresponding to mouse Ser405)-phosphorylated SQSTM1 between *SQSTM1*;*SOD1*^*H46R*^ and *SOD1*^*H46R*^ mice were most comparable (Fig. 8a and b). Further, the ratio of LC3-II/LC3-I in *SQSTM1*;*SOD1*^*H46R*^ mice was lower than that of *SOD1*^*H46R*^ mice (Fig. 8a and d) at onset stage; i.e., 22 week of age. These results combined suggest that overexpression of SQSTM1 does not activate autophagy, but rather accelerates the accumulation of SQSTM1 by possibly hindering the UPS and autophagic degradation in *SOD1*^*H46R*^ mice. Since the accumulation of autophagosome-like structures and SQSTM1-positive aggregates are among the cardinal pathological features in patients with ALS as well as in murine ALS models [18, 23], it is reasonable to assume that overexpression of SQSTM1 enhances mutant SOD1-associated neurotoxicity by further compromising proteostasis, thereby leading to an earlier onset of disease. On the other hand, SBMA-linked toxic entities; e.g., polyglutamine-mediated transcriptional dysregulation [24], may play an indirect role in the regulation of the UPS and/or autophagy, and thus the SQSTM1-associated beneficial effect becomes more tangible in SBMA mice.

Second possibility is that SQSTM1 expressing in cells and tissues other than neurons affects the onset of disease in *SOD1*^*H46R*^ mice. Evidences showing that, in skeletal muscle of a *SOD1*^*G93A*^-expressing ALS mouse model, the UPS is activated prior to the onset of disease, while the autophagic degradation pathway is activated after the onset of disease till at end-stage [36], suggest a possible pathogenic interaction between proteostasis in skeletal muscles and neurodegeneration. Since the expression level of transgene-coded SQSTM1 in skeletal muscle is higher than those in other tissues (Additional file 1: Figure S1b), it is possible that the earlier onset of disease in *SQSTM1*;*SOD1*^*H46R*^ mice is linked to dysregulation of autophagy by SQSTM1 overexpression in skeletal muscles. However, even if that would be the case, the underlying mechanism has yet to be investigated.

Third possibility is that body weight at pre-symptomatic healthy stages affects the onset of disease in *SOD1*^*H46R*^ mice. It has been shown that a high fat food feeding in a mutant TAR DNA-binding protein 43-kD (TARDBP)-expressing ALS mouse model extends their lifespans [37], and that the activation of AMP-activated protein kinase (AMPK), which functions as an energy sensor, results in the extension of lifespans in a mutant SOD1-expressing ALS mouse model [38]. These studies on ALS and energy metabolism suggest that nutritional conditions affect the disease phenotypes in ALS animal models. Interestingly, we revealed that body weight of *SQSTM1* mice tended to be lower than that of WT mice (Fig. 1a and b), and that body weight of female *SQSTM1*;*SOD1*^*H46R*^ mice was significantly lower than that of *SOD1*^*H46R*^ mice throughout their pre-symptomatic period (Fig. 1a). Taken together, it is possible that the reduction of body weight that is associated with SQSTM1 overexpression causes the earlier onset of disease in *SOD1*^*H46R*^ mice. Since loss of SQSTM1 in mice causes hyperphagia that results in obese phenotypes [39], a systemic overexpression of SQSTM1 may conversely induce hypophagia thereby decreasing the body weight in mice. In any cases, pathogenic mechanisms by which overexpression of SQSTM1 accelerates the onset in a *SOD1*^*H46R*^-expressing ALS mouse model remain unknown, and thus further studies are required.

One of the interesting findings in this study is that survival after the onset is slightly extended in *SQSTM1*;*SOD1*^*H46R*^ mice, despite that these mice show earlier onset of disease (Fig. 1). However, we are currently considering that, although the extension of post-onset survival reaches to a statistically significant level, its reliability may still not be high enough to conclude that overexpression of SQSTM1 definitely extends survival, because the prediction of disease onset is solely determined by a single measure; i.e., body weight changes, in this study. Previously, it has been shown that mutant SOD1-mediated toxicities in neurons determine the disease onset, whereas those in astrocytes or microglia regulate the disease progression in ALS mouse models [29, 40]. Based on these findings, it could be hypothesized that mutant SOD1-mediated toxicities in glial cells relative to neurons are decreased by systemic overexpression of SQSTM1 in *SOD1*^*H46R*^ mice. Although quantitative evaluation of GFAP and Iba1 levels by western blotting remained to be performed, immunohistochemical analysis revealed that the glial cells activation in the spinal cord between *SOD1*^*H46R*^ and *SQSTM1*;*SOD1*^*H46R*^ mice were almost comparable (Figs. 4 and 5). Thus, it is fair to assume that such glial contribution may not play a major role in the extension of survival after the onset. On the other hand, we have previously reported that loss of SQSTM1 in *SOD1*^*H46R*^ mice decreases the biochemically-detectable insoluble ubiquitinated proteins in the spinal cord in parallel with the accumulation of ubiquitin-positive inclusions in somal area of motor neurons [25]. Since loss of SQSTM1 exacerbates disease symptoms in *SOD1*^*H46R*^ mice, we consider that the accumulation of biochemically-detectable insoluble ubiquitinated proteins is beneficial and/or protective rather than harmful to motor neurons, while the intrasomal ubiquitin-positive inclusions are poisonous [25]. Indeed, we showed that biochemically-detectable insoluble ubiquitinated proteins were increased in the spinal cord of *SQSTM1*;*SOD1*^*H46R*^ mice at end-stage (Fig. 7a and c). Further, immunohistochemistry revealed that ubiquitin-positive inclusions were exclusively observed in neuropil but not in somal area of motor neurons of *SQSTM1*;*SOD1*^*H46R*^ mice (Figs. 4 and 5). Thus, the effects of loss and overexpression of SQSTM1 on these factors are obviously opposite to each other. Taken together, it is still possible that the insolubilization of poly-ubiquitinated proteins and the exclusion of ubquitinated protein aggregates from neuronal cell body by SQSTM1 are implicated in some neuroprotective roles, particularly, at the stage of disease progression, thereby slowing disease progression by SQSTM1 overexpression in *SOD1*^*H46R*^ mice. However, in order to conclude as such, further studies that includes other methods to determine the onset of disease, such as grip power, the hind-limb reflex, rotarod retention time, and balance-beam test will be required.

## Conclusions

In conclusions, we demonstrated that, unlike in SBMA mice, SQSTM1 served as an onset accelerating factor for SOD1-linked toxicities, at least, in a *SOD1*^*H46R*^-expressing ALS mouse model, implying that SQSTM1 upregulation might not always to be beneficial to the treatment of ALS.

## Additional files

Additional file 1: **Figure S1.** Schema of the transgene construct for *SQSTM1*-tg mouse and the distribution of SQSTM1 in tissues. **a** Schematic diagram of the transgene cassette encoding the C-terminally hemagglutinin (HA)-tagged human *SQSTM1* cDNA. Transgene construct consists of cytomegalovirus enhancer (E), chicken β-actin promoter (Pro), rabbit β-globin splice acceptor (S), full length human *SQSTM1* cDNA (SQSTM1), hemagglutinin tag (HA), and rabbit β-globin poly A (Poly A). **b** Expression and distribution of SQSTM1 in the liver, skeletal muscle, olfactory bulb, cerebral cortex, hippocampus, cerebellum, and spinal cord in wild-type (WT) and *SQSTM1*-tg (SQSTM1) mice. Expression of mouse endogenous SQSTM1 and human SQSTM1 (Endogenous + SQSTM1-HA) was simultaneously detected by western blotting using anti-SQSTM1 antibody. Values shown under each lane were the signal intensities in an arbitrary unit relative to that of the liver in WT mouse. Human SQSTM1 (hSQSTM1-HA) was specifically detected by anti-HA antibody. GAPDH was used as a loading control. (PDF 795 kb)

Additional file 2: **Figure S2.** Whole transverse sectional images of the lumbar spinal cord in Figs. 2, 3 and 4. **a** Whole transverse sectional images of fluorescence Nissl staining in the lumbar spinal cords in Fig. 2. **b** Whole transverse sectional images of double immunostaining with Ubiquitin (green) and SQSTM1 (red) in the lumbar spinal cord in Fig. 3. **c** Whole transverse sectional images of double immunostaining with MAP2 (green) and Ubiquitin (red), Ubiquitin (green) and GFAP (red), and Ubiquitin (green) and Iba1 (red) in the lumbar spinal cord in Fig. 4. **a-c** Scale bars = 300 μm. **b, c** The nuclei were counterstained with DAPI (blue). (PDF 7238 kb)

Additional file 3: **Figure S3.** SQSTM1-HA positive aggregates in the anterior horn of lumbar spinal cord. **a-c** Representative images of double immunostaining with Ubiquitin (green) and HA (SQSTM1-HA; red) in the lumbar spinal cord (L4–5) from wild-type (WT), *SQSTM1* (SQSTM1), *SOD1*^*H46R*^ (H46R) and *SQSTM1*;*SOD1*^*H46R*^ (SQSTM1;H46R) mice at 16 weeks of age (wk) (**a**), 22 wk. (**b**), and end-stage (H46R and SQSTM1;H46R) or 28 wk. (WT and SQSTM1) (**c**). The nuclei were counterstained with DAPI (blue). Scale bars = 50 μm. Ubiquitin-positive aggregates and SQSTM1-HA aggregates were observed in the anterior horn of *SQSTM1*;*SOD1*^*H46R*^ (SQSTM1;H46R) mice at 22 wk. and end-stage. Arrows indicate that ubiquitin-positive aggregates colocalizing with SQSTM1-HA. (PDF 1718 kb)

Additional file 4: **Figure S4.** Immunoblot-images used for the quantitative analysis in Figs. 6a and Fig. 7. The immunoblots of (**a**) soluble SOD1, (**b**) insoluble SOD1 monomer (Mono), (**c**) insoluble high-molecular weight (HMW) SOD1, (**d**) soluble SQSTM1, (**e**) insoluble SQSTM1, (**f**) soluble poly-ubiquitinated proteins, (**g**) insoluble poly-ubiquitinated proteins, (**h**) soluble GAPDH, and (**i**) insoluble actin were analyzed for the quantitative analysis (Figs. 6a and 7). The spinal cord from wild-type (WT), *SQSTM1* (SQSTM1), *SOD1*^*H46R*^ (H46R), and *SQSTM1*;*SOD1*^*H46R*^ (SQSTM1;H46R) mice at 16 weeks of age (wk), 22 wk., and end-stage (H46R and SQSTM1;H46R) or 28 wk. (WT and SQSTM1) were used. C (control sample) used as internal control indicates soluble and insoluble fractions from 22 week-old *SQSTM1;SOD1*^*H46R*^ mouse. Asterisk represents the measured area of poly-ubiquitinated proteins. (PDF 1973 kb)

Additional file 5: **Figure S5.** Immunoblot-images used for the quantitative analysis in Fig. 6b. The immunoblots of (**a**) soluble misfolded SOD1 and GAPDH, (**b**) insoluble misfolded SOD1 and actin were analyzed for the quantitative analysis (Fig. 6b). The spinal cord from *SOD1*^*H46R*^ (H46R), and *SQSTM1*;*SOD1*^*H46R*^ (SQSTM1;H46R) mice at 16 weeks of age (wk), 22 wk., and end-stage were used. (PDF 444 kb)

Additional file 6: **Figure S6.** Immunoblot-images used for the quantitative analysis in Fig. 8. The immunoblots of (**a**) soluble and (**b**) insoluble Ser403(human)/Ser405(mouse)-phosphorylated SQSTM1 (p-S403/405), (**c**) soluble and (**d**) insoluble Ser349(human)/Ser351(mouse)-phosphorylated SQSTM1 (p-S349/351), (**e**) soluble LC3, (**f**) insoluble LC3, (**g**) soluble NQO1, and (**h**) insoluble NQO1 were analyzed for the quantitative analysis (Fig. 7). The spinal cord from wild-type (WT), *SQSTM1* (SQSTM1), *SOD1*^*H46R*^ (H46R), and *SQSTM1*;*SOD1*^*H46R*^ (SQSTM1;H46R) mice at 16 weeks of age (wk), 22 wk., and end-stage (H46R and SQSTM1;H46R) or 28 wk. (WT and SQSTM1) were used. C (control sample) used as internal control indicates soluble and insoluble fractions from 22 week-old *SQSTM1;SOD1*^*H46R*^ mouse. (PDF 607 kb)

## Abbreviations

ALS: Amyotrophic lateral sclerosis
AMPK: Adenosine mono-phosphate activated protein kinase
ANOVA: Analysis of variance
BPB: Bromophenol blue
CAG: (C) the cytomegalovirus (CMV) early enhancer element, (A) the promoter, the first exon and the first intron of chicken beta-actin gene, and (G) the splice acceptor of the rabbit beta-globin gene
DAPI: 4′,6-diamidino-2-phenylindole dihydrochloride
FTD: Frontotemporal dementia
GAPDH: Glyceraldehyde 3-phosphate dehydrogenase
GFAP: Glial fibrillary acidic protein
HA: Hemagglutinin
HRP: Horseradish peroxidase
Iba1: Ionized calcium binding adaptor molecule 1
KEAP1: Kelch-like ECH-associated protein 1
MAP1LC3/LC3: Microtubule-associated protein 1 light chain 3
MAP2: Microtubule-associated protein 2
MND: Motor neuron disease
NFE2L2/Nrf2: Nuclear factor related erythroid 2-related factor 2
NGS: Normal goat serum
NQO1: NAD(P)H dehydrogenase [quinone] 1
PAGE: Polyacrylamide gel electrophoresis
PB: Phosphate buffer
PBS: Phosphate-buffered saline
PFA: Paraformaldehyde
PVDF: Polyvinylidene difluoride
RT: Room temperature
SBMA: Spinal and bulbar muscular atrophy
SDS: Sodium dodecyl sulfate
SQSTM1: Sequestosome 1/p62
TARDBP: TAR DNA-binding protein 43-kD
tg: Transgenic
UPS: Ubiquitin-proteasome system
WT: Wild-type
