# Supplementary figures and images for: Systemic overexpression of SQSTM1/p62 accelerates disease onset in a SOD1H46R-expressing ALS mouse model
Source: Mol Brain. 2018 May 29;11:30. doi: 10.1186/s13041-018-0373-8 (PMC5975400; doi:10.1186/s13041-018-0373-8)

**a**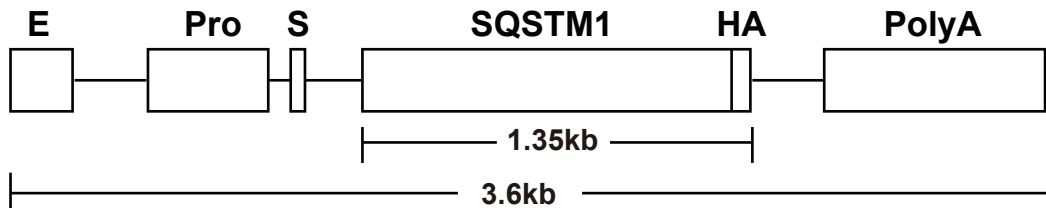**b**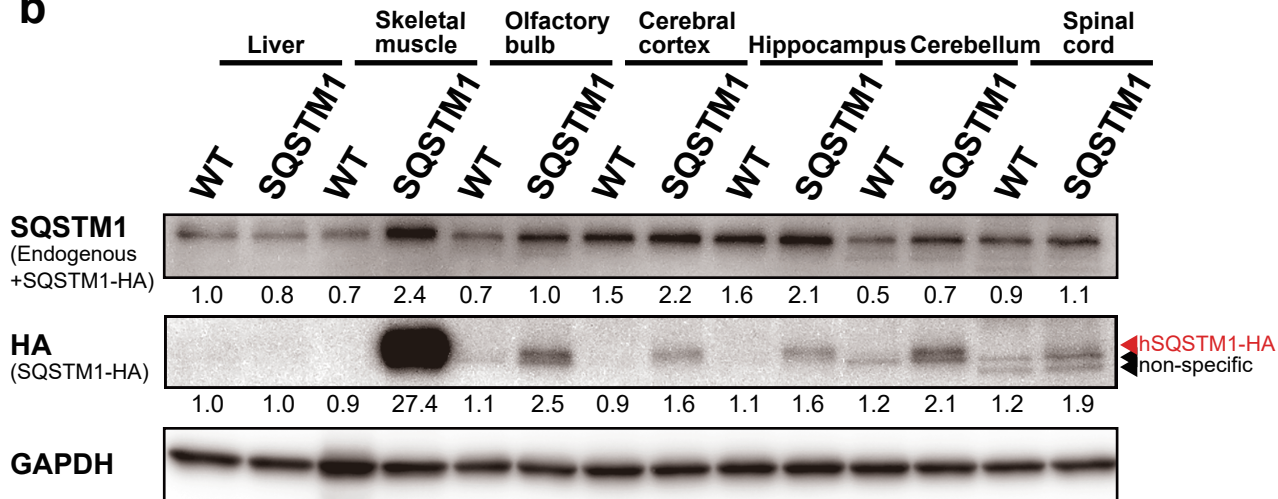

Supplement: Supplementary file 1 — Figure S1. Schema of the transgene construct for SQSTM1-tg mouse and the distribution of SQSTM1 in tissues. a Schematic diagram of the transgene cassette encoding the C-terminally hemagglutinin (HA)-tagged human SQSTM1 cDNA. Transgene construct consists of cytomegalovirus enhancer (E), chicken β-actin promoter (Pro), rabbit β-globin splice acceptor (S), full length human SQSTM1 cDNA (SQSTM1), hemagglutinin tag (HA), and rabbit β-globin poly A (Poly A). b Expression and distribution of SQSTM1 in the liver, skeletal muscle, olfactory bulb, cerebral cortex, hippocampus, cerebellum, and spinal cord in wild-type (WT) and SQSTM1-tg (SQSTM1) mice. Expression of mouse endogenous SQSTM1 and human SQSTM1 (Endogenous + SQSTM1-HA) was simultaneously detected by western blotting using anti-SQSTM1 antibody. Values shown under each lane were the signal intensities in an arbitrary unit relative to that of the liver in WT mouse. Human SQSTM1 (hSQSTM1-HA) was specifically detected by anti-HA antibody. GAPDH was used as a loading control. (PDF 795 kb) [file 13041_2018_373_MOESM1_ESM.pdf]

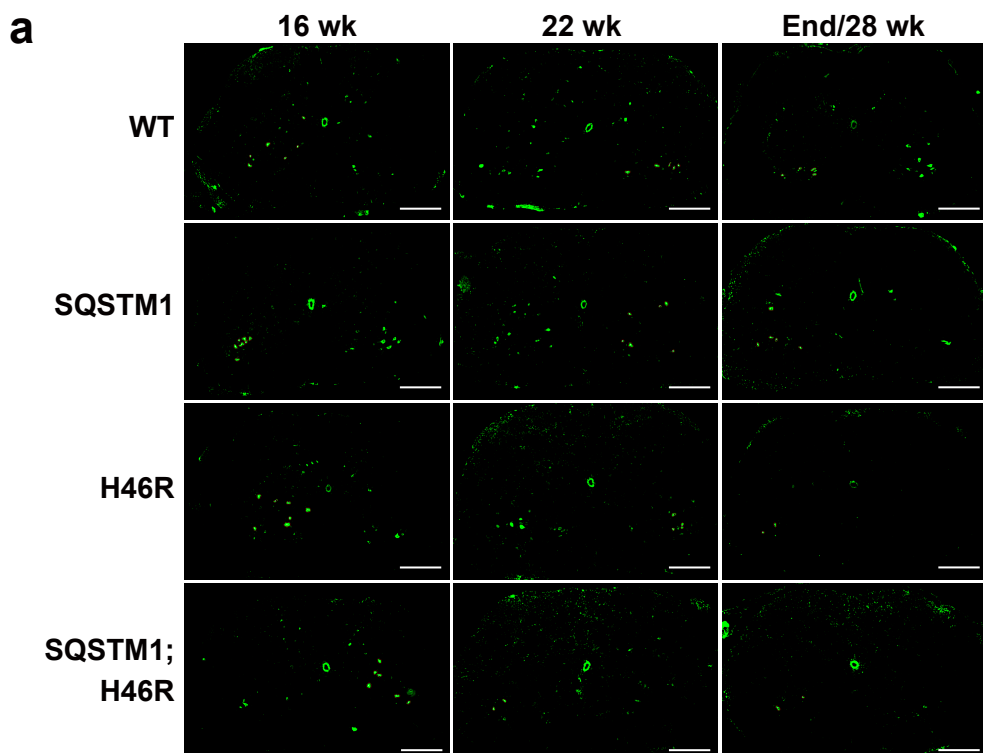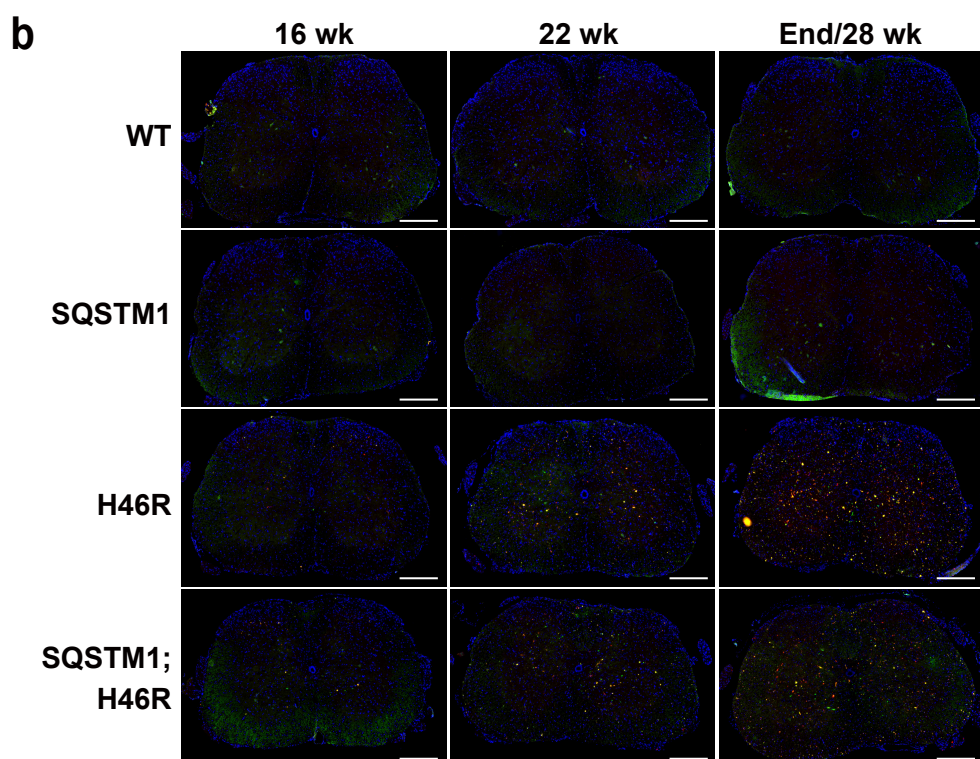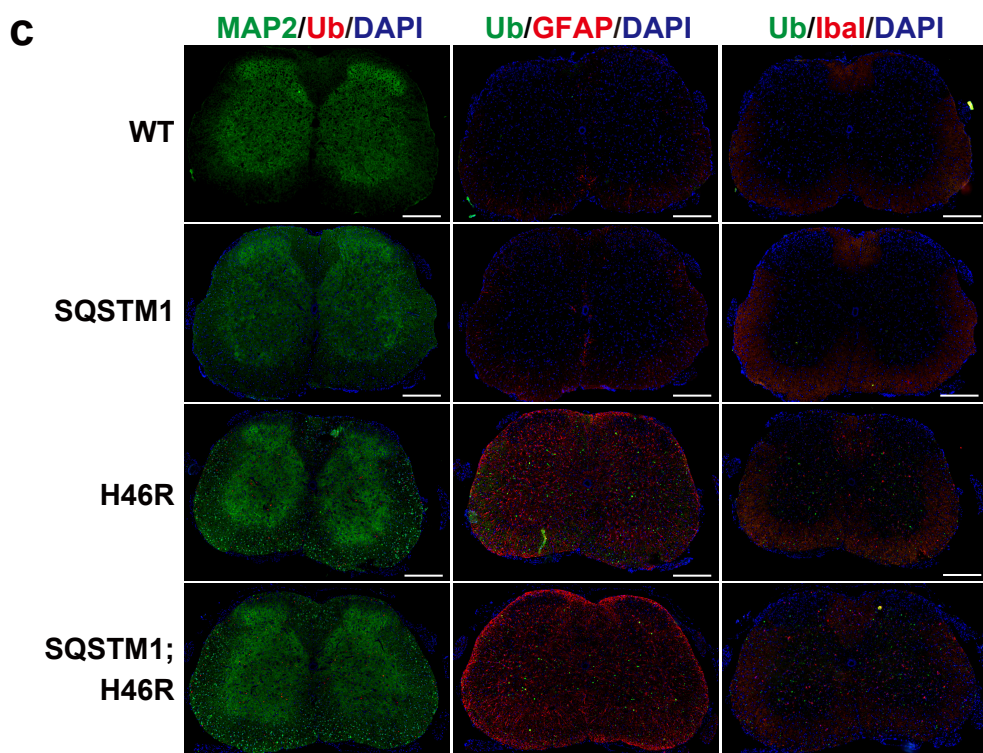

Supplement: Supplementary file 2 — Figure S2. Whole transverse sectional images of the lumbar spinal cord in Figs. 2, 3 and 4. a Whole transverse sectional images of fluorescence Nissl staining in the lumbar spinal cords in Fig. 2. b Whole transverse sectional images of double immunostaining with Ubiquitin (green) and SQSTM1 (red) in the lumbar spinal cord in Fig. 3. c Whole transverse sectional images of double immunostaining with MAP2 (green) and Ubiquitin (red), Ubiquitin (green) and GFAP (red), and Ubiquitin (green) and Iba1 (red) in the lumbar spinal cord in Fig. 4. a-c Scale bars = 300 μm. b, c The nuclei were counterstained with DAPI (blue). (PDF 7238 kb) [file 13041_2018_373_MOESM2_ESM.pdf]

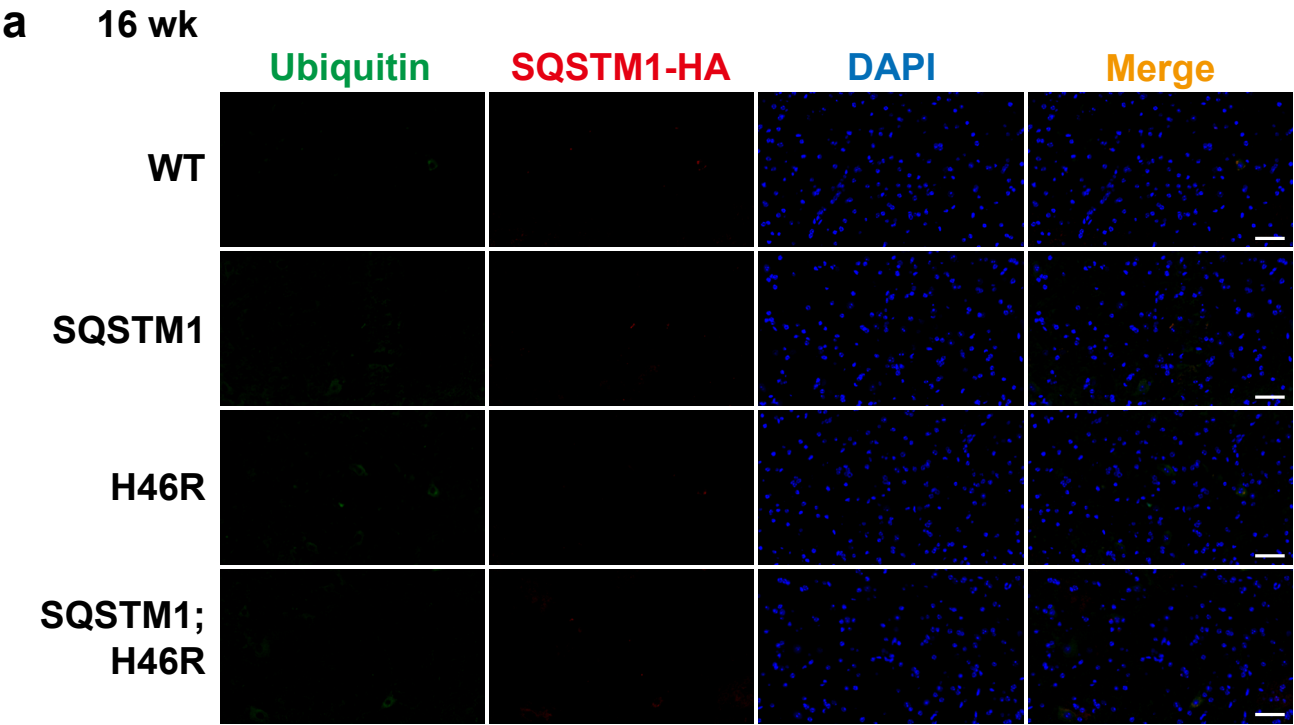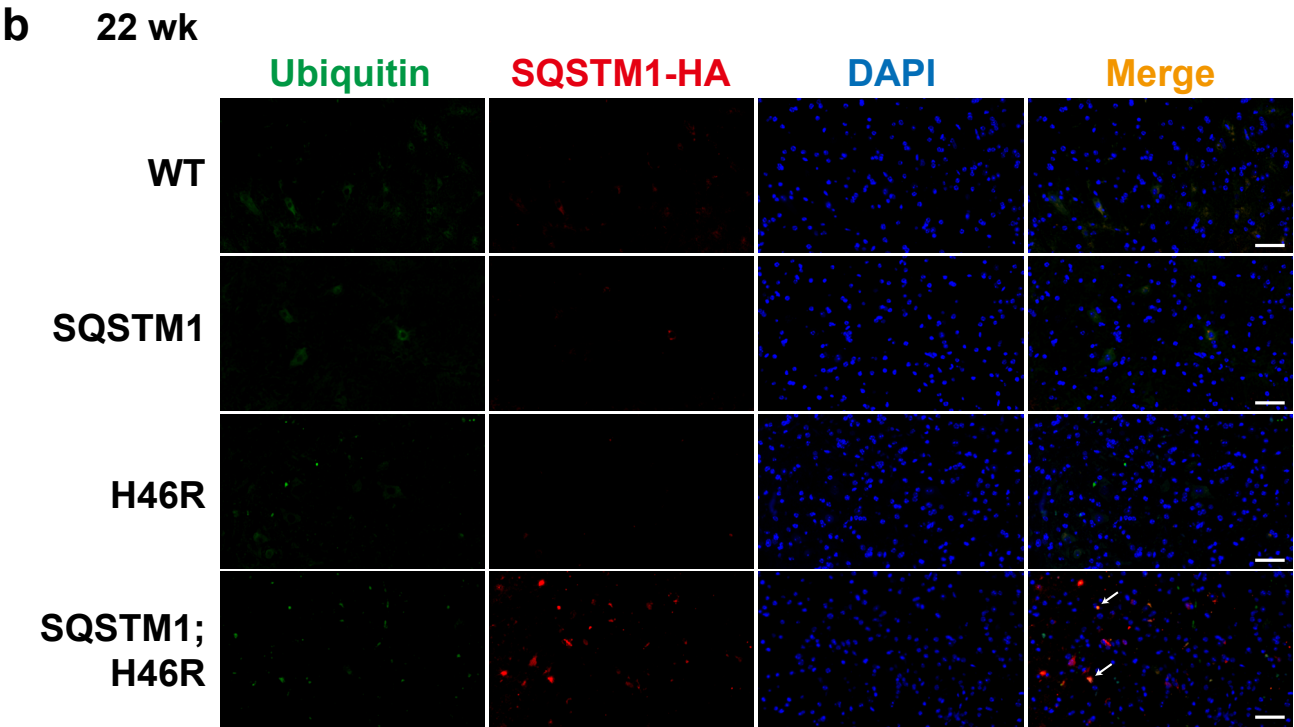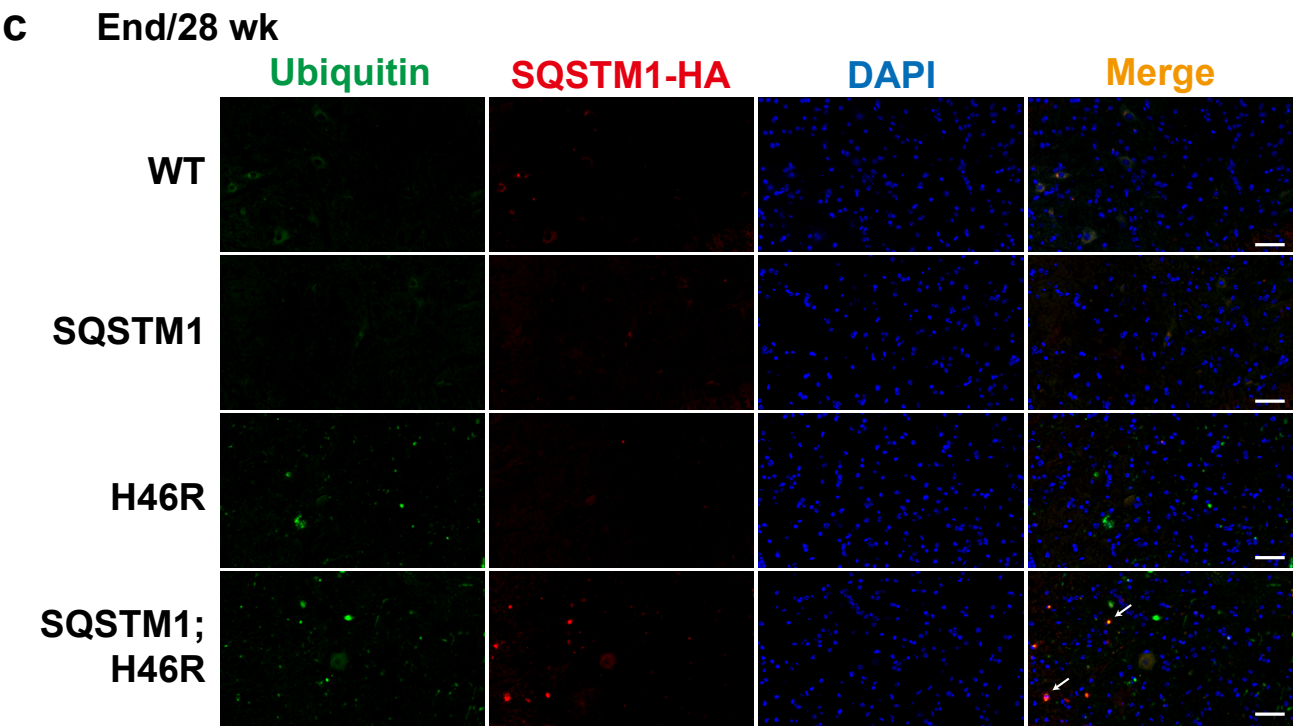

Supplement: Supplementary file 3 — Figure S3. SQSTM1-HA positive aggregates in the anterior horn of lumbar spinal cord. a-c Representative images of double immunostaining with Ubiquitin (green) and HA (SQSTM1-HA; red) in the lumbar spinal cord (L4–5) from wild-type (WT), SQSTM1 (SQSTM1), SOD1H46R (H46R) and SQSTM1;SOD1H46R (SQSTM1;H46R) mice at 16 weeks of age (wk) (a), 22 wk. (b), and end-stage (H46R and SQSTM1;H46R) or 28 wk. (WT and SQSTM1) (c). The nuclei were counterstained with DAPI (blue). Scale bars = 50 μm. Ubiquitin-positive aggregates and SQSTM1-HA aggregates were observed in the anterior horn of SQSTM1;SOD1H46R (SQSTM1;H46R) mice at 22 wk. and end-stage. Arrows indicate that ubiquitin-positive aggregates colocalizing with SQSTM1-HA. (PDF 1718 kb) [file 13041_2018_373_MOESM3_ESM.pdf]

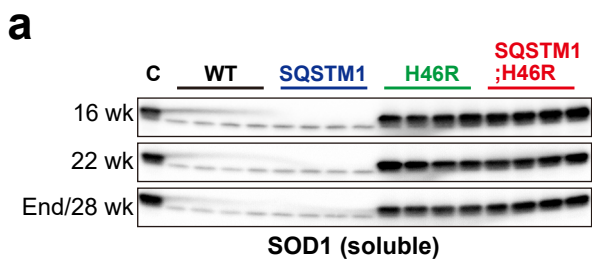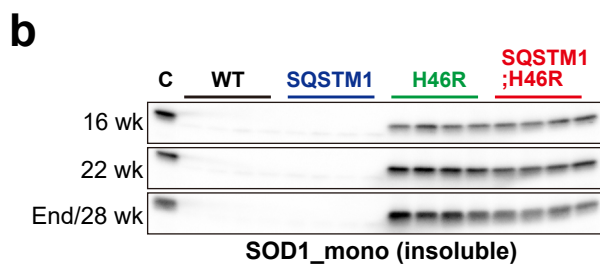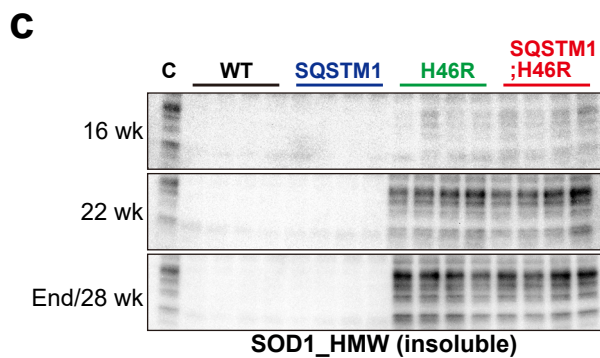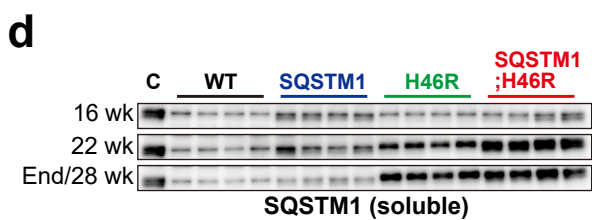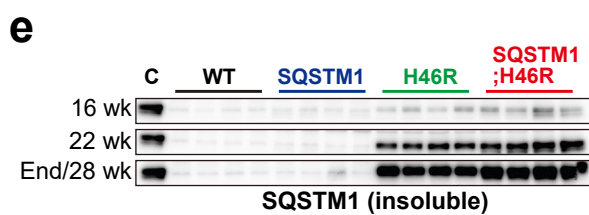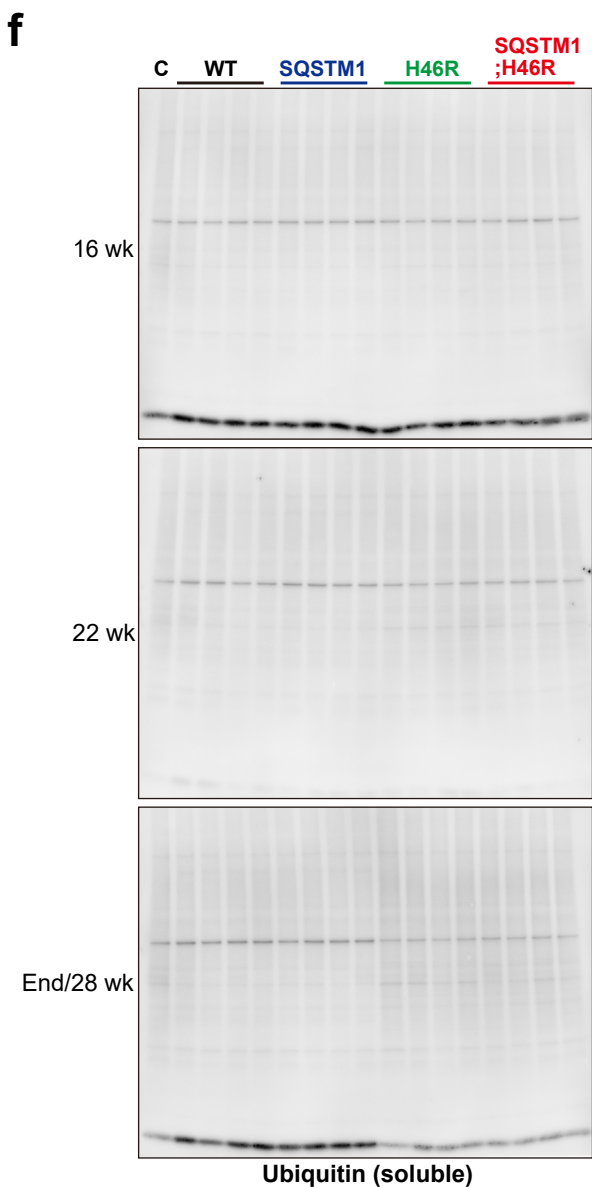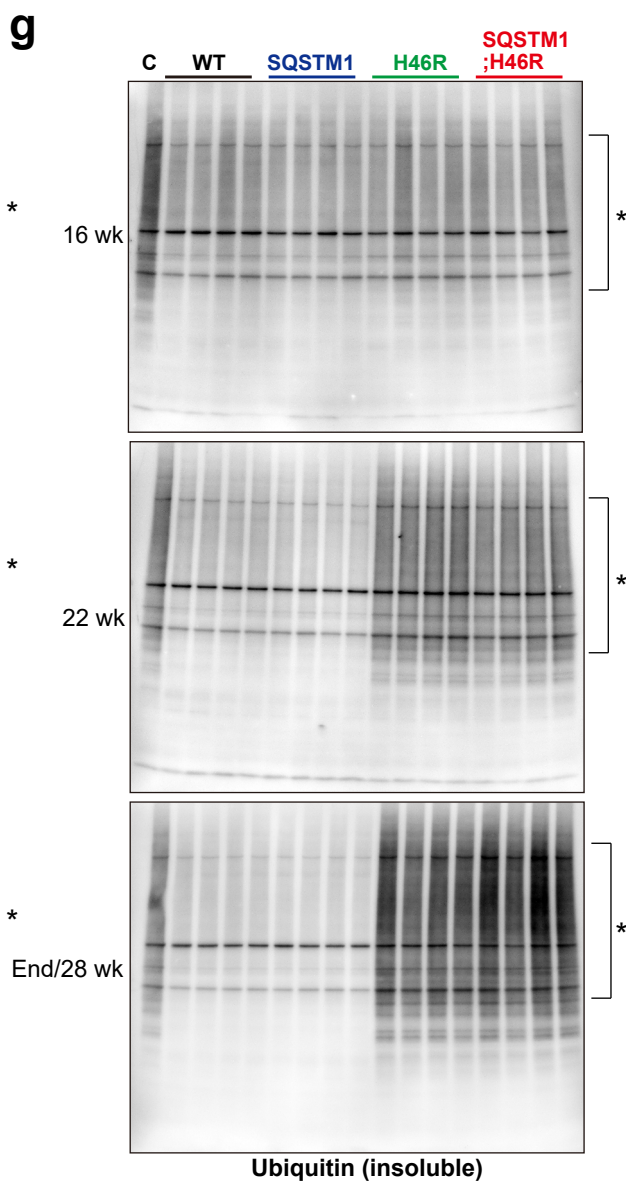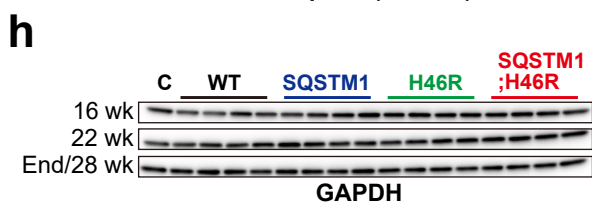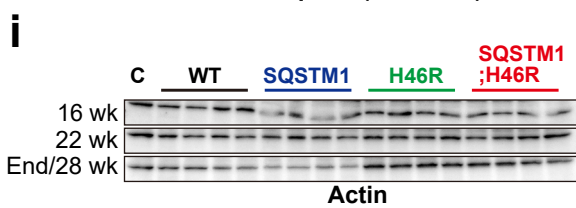

Supplement: Supplementary file 4 — Figure S4. Immunoblot-images used for the quantitative analysis in Figs. 6a and Fig. 7. The immunoblots of (a) soluble SOD1, (b) insoluble SOD1 monomer (Mono), (c) insoluble high-molecular weight (HMW) SOD1, (d) soluble SQSTM1, (e) insoluble SQSTM1, (f) soluble poly-ubiquitinated proteins, (g) insoluble poly-ubiquitinated proteins, (h) soluble GAPDH, and (i) insoluble actin were analyzed for the quantitative analysis (Figs. 6a and 7). The spinal cord from wild-type (WT), SQSTM1 (SQSTM1), SOD1H46R (H46R), and SQSTM1;SOD1H46R (SQSTM1;H46R) mice at 16 weeks of age (wk), 22 wk., and end-stage (H46R and SQSTM1;H46R) or 28 wk. (WT and SQSTM1) were used. C (control sample) used as internal control indicates soluble and insoluble fractions from 22 week-old SQSTM1;SOD1H46R mouse. Asterisk represents the measured area of poly-ubiquitinated proteins. (PDF 1973 kb) [file 13041_2018_373_MOESM4_ESM.pdf]

**a**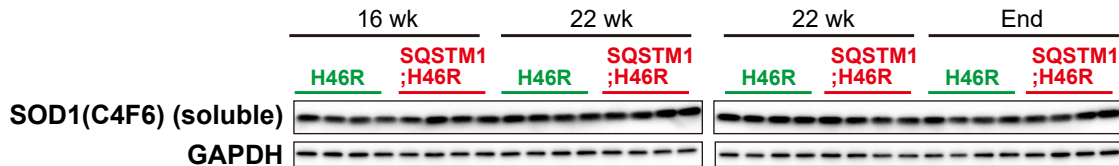**b**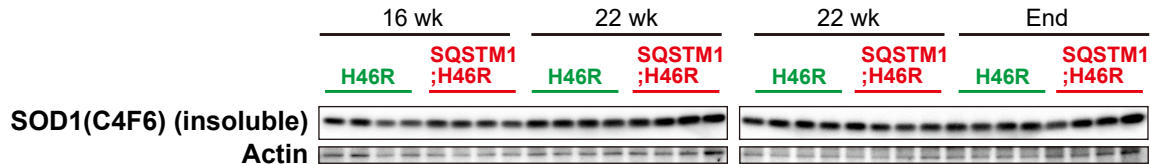

Supplement: Supplementary file 5 — Figure S5. Immunoblot-images used for the quantitative analysis in Fig. 6b. The immunoblots of (a) soluble misfolded SOD1 and GAPDH, (b) insoluble misfolded SOD1 and actin were analyzed for the quantitative analysis (Fig. 6b). The spinal cord from SOD1H46R (H46R), and SQSTM1;SOD1H46R (SQSTM1;H46R) mice at 16 weeks of age (wk), 22 wk., and end-stage were used. (PDF 444 kb) [file 13041_2018_373_MOESM5_ESM.pdf]

**a**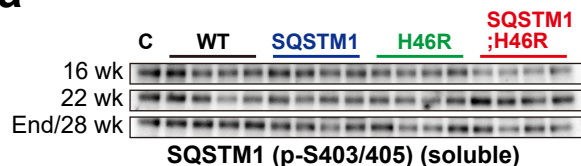**b**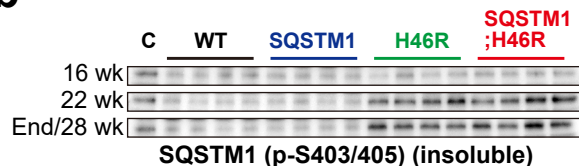**c**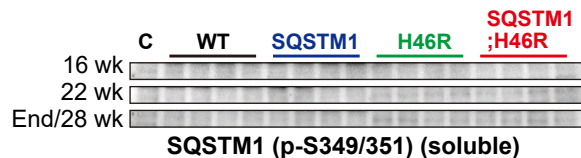**d**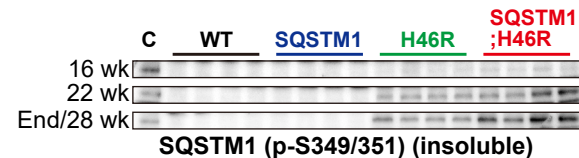**e**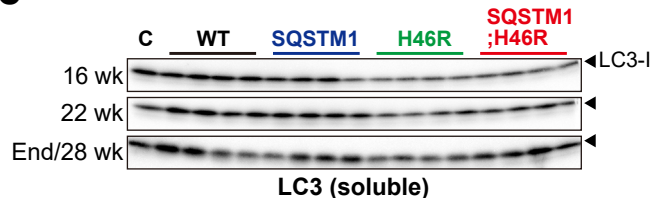**f**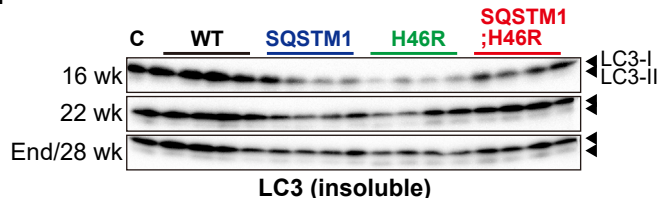**g**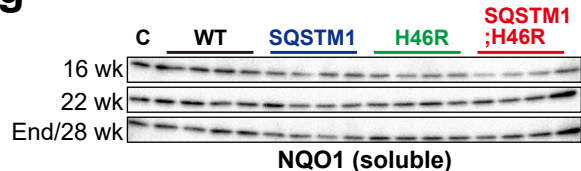**h**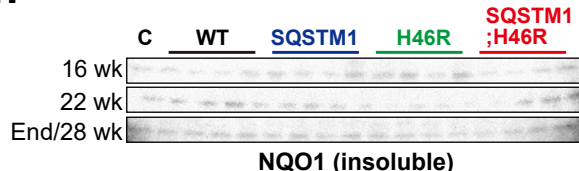

Supplement: Supplementary file 6 — Figure S6. Immunoblot-images used for the quantitative analysis in Fig. 8. The immunoblots of (a) soluble and (b) insoluble Ser403(human)/Ser405(mouse)-phosphorylated SQSTM1 (p-S403/405), (c) soluble and (d) insoluble Ser349(human)/Ser351(mouse)-phosphorylated SQSTM1 (p-S349/351), (e) soluble LC3, (f) insoluble LC3, (g) soluble NQO1, and (h) insoluble NQO1 were analyzed for the quantitative analysis (Fig. 7). The spinal cord from wild-type (WT), SQSTM1 (SQSTM1), SOD1H46R (H46R), and SQSTM1;SOD1H46R (SQSTM1;H46R) mice at 16 weeks of age (wk), 22 wk., and end-stage (H46R and SQSTM1;H46R) or 28 wk. (WT and SQSTM1) were used. C (control sample) used as internal control indicates soluble and insoluble fractions from 22 week-old SQSTM1;SOD1H46R mouse. (PDF 607 kb) [file 13041_2018_373_MOESM6_ESM.pdf]
